# An improved mountain gazelle optimizer based on chaotic map and spiral disturbance for medical feature selection

**DOI:** 10.1371/journal.pone.0307288

**Published:** 2024-07-16

**Authors:** Ying Li, Yanyu Geng, Huankun Sheng

**Affiliations:** 1 College of Computer Science and Technology, Jilin University, Changchun, People’s Republic of China; 2 Key Laboratory of Symbolic Computation and Knowledge Engineering of Ministry of Education, Jilin University, Changchun, People’s Republic of China; Firat Universitesi, TÜRKIYE

## Abstract

Feature selection is an important solution for dealing with high-dimensional data in the fields of machine learning and data mining. In this paper, we present an improved mountain gazelle optimizer (IMGO) based on the newly proposed mountain gazelle optimizer (MGO) and design a binary version of IMGO (BIMGO) to solve the feature selection problem for medical data. First, the gazelle population is initialized using iterative chaotic map with infinite collapses (ICMIC) mapping, which increases the diversity of the population. Second, a nonlinear control factor is introduced to balance the exploration and exploitation components of the algorithm. Individuals in the population are perturbed using a spiral perturbation mechanism to enhance the local search capability of the algorithm. Finally, a neighborhood search strategy is used for the optimal individuals to enhance the exploitation and convergence capabilities of the algorithm. The superior ability of the IMGO algorithm to solve continuous problems is demonstrated on 23 benchmark datasets. Then, BIMGO is evaluated on 16 medical datasets of different dimensions and compared with 8 well-known metaheuristic algorithms. The experimental results indicate that BIMGO outperforms the competing algorithms in terms of the fitness value, number of selected features and sensitivity. In addition, the statistical results of the experiments demonstrate the significantly superior ability of BIMGO to select the most effective features in medical datasets.

## I. Introduction

With the continuous development of medical informatics, the amount of medical data is growing rapidly. Medical data are a general term for data and information from multiple fields such as consultation services, disease prevention, health checkups, etc. These data mainly include: electronic medical records, medical images, laboratory data, sign indicator data and personal health data. Medical data is a crucial diagnostic basis for doctors, providing more comprehensive treatment clues and assisting doctors in providing more accurate diagnoses. Disease diagnosis is the cornerstone of prevention and treatment and can be used to determine the type, characteristics, and severity of diseases through medical data analysis and early symptom detection, providing the basis for early intervention and treatment. The diagnostic accuracy directly affects the success rate of disease treatment. The improvement of the accuracy rate can not only effectively improve the cure success rate, survival rate, survival cycle and quality of life of patients but also reduce the medical cost of patients.

However, current medical data face problems such as large data volume, multiple data types, high data dimensionality, high value but low value density, and real-time nature [1]. These challenges lead to high time costs, low diagnostic accuracy, and a reliance on empirical knowledge in disease diagnosis and research [2], hindering patient recovery, survival rate, and quality of life improvements and healthcare cost reductions [3].

In this context, integrating machine learning techniques for medical diagnosis is emerging as a significant research trend [4, 5]. Nonetheless, raw medical data contain numerous irrelevant and redundant features [6], which not only obstruct data analysis but also lead to the ’curse of dimensionality’ [7]. Consequently, extracting essential information effectively from raw data is important for enhancing both the accuracy and efficiency of diagnoses.

Feature selection (FS) has always been a critical research domain in machine learning. Identifying the most relevant and effective feature subsets is the objective of FS [8, 9]. In the domain of medical data processing, FS is particularly important, aiding in selecting the most representative and useful features and thereby simplifying the model and enhancing the efficiency and accuracy of data processing. Additionally, FS improves the model interpretability, enabling doctors to understand the decision-making process, which in turn increases their trust in the model. Finally, robust models can be established by eliminating irrelevant and redundant features [10].

However, searching within the feature subspace to identify useful feature subsets is an NP-hard optimization problem [11–13]. All search methods can be broadly categorized into three types: complete search algorithms, heuristic search algorithms, and random search algorithms [14]. The first type of algorithm is rarely used because it requires considerable computing power and is easily affected by changes in the size of the data. Heuristic search algorithms generally have moderate search capabilities. They are prone to becoming trapped in local optima and cannot effectively handle the problem of combination explosion in feature subset solution spaces [15]. Random search algorithms, represented by metaheuristic algorithms, use stochastic methods to obtain feature subsets, allowing for a larger search space and effectively avoiding local optima [16, 17]. Metaheuristics are primarily split into single-solution-based and population-based algorithms [18]. The former works by optimizing a single solution. In contrast, the latter creates a group of solutions, termed a ’population’, in each iteration. This approach is more effective at avoiding local optima [19]. Population-based metaheuristic methods can be further divided into evolution-based algorithms, human-based algorithms, physics-based algorithms, sports-based algorithms, light-based intelligent algorithms, and swarm intelligence algorithms [20, 21].

Inspired by natural biological populations, swarm intelligence algorithms offer innovative solutions for subspace searching [22]. Key examples of swarm intelligence algorithms include particle swarm optimization (PSO) [23], ant colony optimization (ACO) [24], cuckoo optimization algorithm (COA) [25], bat algorithm (BA) [26], gray wolf optimizer (GWO) [27], whale optimization algorithm (WOA) [28], salp swarm algorithm(SSA) [29], nutcracker optimizer algorithm (NOA) [30], manta ray foraging optimization (MRFO) [31], African vulture optimization algorithm (AVOA) [32], Harris hawks optimization (HHO) [33], and more.

These algorithms process medical data effectively via distributed computing. Information-sharing mechanisms also significantly improve the model efficiency and adaptability. Additionally, their inherent flexibility and robustness ensure stable performance, even in the presence of individual errors or failures. Importantly, swarm intelligence algorithms excel at preventing premature convergence and achieve superior optimization precision through collaborative decision-making and strategic search methodologies.

Swarm intelligence algorithms have achieved significant success in medical data FS. However, it is difficult for these algorithms to balance exploration and exploitation, maintain population diversity, maintain convergence, and adjust parameters [34, 35]. Moreover, many studies using swarm intelligence algorithms for medical data feature selection address simple and limited problems, targeting only specific diseases or datasets, with a generalizability that is not better adapted to today’s rapidly evolving medical data needs. In addition, some algorithms are inefficient for large-scale data. Therefore, given the characteristics of medical data, such as the variability and instability of the data and feature volume [36], more flexible and adaptive processing methods are needed.

The introduction of a novel algorithm named the mountain gazelle optimizer (MGO) is not affected by parameter settings because it is parameter-free [37–39]. In addition, the algorithm perfectly balances exploration and exploitation by using four different mechanisms at all optimization stages. Moreover, since the MGO uses many finite vectors, it has an excellent ability to escape from local optima and can explore all optimization spaces. In addition, according to the experimental results of Abdollahzadeh et al., the MGO algorithm has a strong ability to solve continuous problems, with very good results when both the population size and dimensionality change.

However, the MGO still has certain limitations in terms of solution diversity, local search ability, and escaping local optima. Moreover, the current MGO algorithm is mainly used to solve continuous problems, and there is no binary version for solving feature selection problems. This situation prompted us to improve the MGO for these problems and apply it to feature selection tasks.

In this study, we first propose an improved mountain gazelle algorithm named IMGO, which uses an iterative chaotic map with infinite collapses (ICMIC) to initialize the gazelle population and nonlinear factors to control the coefficient vectors, includes a spiral perturbation mechanism to perturb the position of individuals and performs a depth search of the neighborhood of the optimal individuals. To verify the improved performance of this algorithm, it is evaluated on 23 benchmark functions, and its superiority is demonstrated in comparison experiments with the original algorithm and 8 well-known and newly proposed metaheuristic algorithms, namely, the WOA, PSO, GWO, marine predator algorithm (MPA) [40], NOA, Kepler optimization algorithm (KOA) [41], SSA and BA. The binary version of IMGO (BIMGO) is then developed to handle the feature selection task. The proposed BIMGO algorithm is evaluated on 16 medical datasets and compared with the binary versions of the eight metaheuristic algorithms mentioned above. The experimental results show that the proposed BIMGO algorithm outperforms the comparison algorithms in terms of the fitness value, number of selected features, and convergence. In addition, the use of the 5% Wilcoxon rank-sum test verifies that the BIMGO algorithm performs significantly better than the competing algorithms on most of the datasets.

The main contributions of this paper are summarized as follows:

- For the problem of low population diversity of the original algorithm, in the population initialization stage, ICMIC chaotic mapping is used instead of the original random initialization to improve the diversity of the population.
- By introducing a nonlinear control factor, the global search in the early stage of the algorithm and the local search in the late stage of the algorithm are enhanced, and the search efficiency of the algorithm is improved.
- To enhance the local search ability of the algorithm and eliminate local traps, a spiral perturbation mechanism is adopted to perturb the position of individual gazelles during the iteration process.
- The algorithm searches for the neighborhood of the optimal individual at the later stage of each iteration, effectively enhancing the development capability of the algorithm.
- A binary version of the IMGO is developed to apply the proposed algorithm to the feature selection task, which is ideal for dealing with feature selection in medical data.

The remainder of this paper is structured as follows. The second section describes related progress on using swarm intelligence algorithms to analyze medical data. The MGO algorithm and the proposed IMGO and BIMGO algorithms are presented in Section III. In the fourth section, the IMGO is evaluated on 23 benchmark functions. Furthermore, the applicability of the BIMGO for extracting effective features from medical data is verified. The last section discusses the conclusions and future research directions of this research.

## II. Related works

Swarm intelligence algorithms have shown significant advantages in accomplishing feature selection tasks represented by medical data through mechanisms such as distributed computing, information sharing, and collaborative decision making. This section reviews related works on the use of swarm intelligence algorithms to solve the problem of medical data analysis, which ranges from early research on simple identification of important factors for diseases to more recent research on medical diagnosis aids.

Chen et al. [42] illustrated a combination of PSO and the 1-nearest neighbor (1-NN) mechanism, which can effectively identify important factors in patients with obstructive sleep apnea (OSA). Lin et al. [43] combined the endocrine-based PSO algorithm with the artificial bee colony (ABC) algorithm and used support vector machines (SVMs) to perform sorting on specific medical datasets. Brahim Sahmad et al. [44] integrated the binary firefly algorithm, quickly identifying high-quality solutions and significantly improving the classification accuracy on medical datasets. Anter et al. [45] introduced a hybrid crow search optimization algorithm that combines chaos theory with the fuzzy c-means algorithm. The performance of this hybrid approach on medical datasets demonstrated its effectiveness and stability. Sahlol et al. [46] combined fractional order and the marine predator algorithm (MPA), which achieved high classification accuracy on a COVID-19 dataset. Asghari Varzaneh et al. [47] applied the horse herd optimization algorithm (HOA) to predict the intubation risk of hospitalized patients with COVID-19, efficiently identifying critical predictive factors for better performance. Nadimi Shahraki et al. [48] deployed a binary version of the quantum-based avian navigation optimizer algorithm (BQANA) with a threshold method for high-dimensional medical datasets. The method outperforms nine well-known binary metaheuristic algorithms in optimal feature subset selection. Elgamal et al. [49] suggested an improved reptile search algorithm (IRSA) by incorporating chaos theory and simulated annealing. This method effectively improves the search capability, performing better than the original algorithm and comparison methods on medical datasets. Wang et al. [50] introduced SVM-MPA, a novel combination of the MPA with an SVM. They constructed an effective subject-independent anterior cruciate ligament defect detection model to provide an accurate preoperative auxiliary testing method for the clinical diagnosis of anterior cruciate ligament (ACL) deficiency. Finally, Mohammad H. Nadimi-Shahraki [51] enhanced the WOA with the suggested mechanism and search strategies. They proposed the E-WOA and BE-WOA as binary versions verified on a medical disease dataset. The test results showed that the E-WOA outperformed the latest optimization methods. This algorithm was also successfully applied to diagnose COVID-19, providing a feasible model for diagnostic medical treatment. Braik et al. [52] proposed three methods based on the Capuchin search algorithm, ECSA, PCSA, and SCSA, combined the binary versions of these three algorithms with a k-nearest neighbor (KNN) classifier, and demonstrated their performance on a medical dataset. To address the challenge of rapidly increasing glaucoma infections, Singh et al. [53] proposed a hybrid algorithm based on the emperor penguin optimization algorithm and bacterial foraging optimization to extract effective features from retinal fundus baseline images, minimizing the number of features while improving the classification accuracy. This method can assist overworked ophthalmologists and prevent individuals from losing vision. To address the problems of slow convergence and imbalance between exploration and exploitation with the hunger games search (HGS), Hashim et al. [54] proposed an improved version of the HGS named mHGS, which was able to solve the feature selection problem well for Parkinson’s disease phonation datasets. Neggaz et al. [55] proposed an enhanced variant of the manta ray foraging optimizer (MRFO), MRFOSCA, using trigonometry operators inspired by the sine cosine algorithm (SCA), effectively improving the problem of convergence to local minima. This approach was used to solve the feature selection problem represented by medical datasets.

Related studies have shown that various swarm intelligence algorithms have been used to select important features from medical data to assist in data analysis, as shown in Table 1. However, the aforementioned swarm intelligence algorithms have some limitations when dealing with medical datasets. First, some studies only target specific and single medical data, without the universality of analyzing medical data. Second, some studies have focused on low-dimensional datasets and may not have had the ability to analyze current high- and ultrahigh-dimensional medical data. Again, there are issues of unbalanced search strategies and low population diversity. Finally, some studies only involve medical data and are not specifically focused on medical data, and the evaluation criteria used do not account for the characteristics of medical data.

**Table 1:** Related researches.

Refs.	Author	Year	Optimization algorithm	Classifier/Architecture	Datasets	Dimension
[42]	Chen et al.	2012	PSO	KNN	An actual case from the Sleep Center of the Chang Gung Memorial Hospital in Taipei, 8 UCI medical datasets	3–44
[43]	Lin et al.	2015	EPSO_ABC	SVM	6 UCI medical datasets	7–22
[44]	Sahmad et al.	2018	BFA	SVM	11 UCI medical datasets	6–2000
[45]	Anter et al.	2020	CFCSA	KNN	1 CT liver tumor diagnosis data set, 9 UCI medical datasets	7–112
[46]	Sahlol et al.	2020	FO-MPA	CNN	2 COVID-19 X-ray datasets	51000
[47]	Varzaneh et al.	2021	HOA	KNN	COVID-19 hospital registration database	54
[48]	Nadimi-Shahraki et al.	2022	BQANA	KNN	10 UCI medical datasets	8–10509
[49]	Elgamal et al.	2022	IRSA	KNN	20 UCI medical datasets	10–279
[50]	Wang et al.	2022	MPA	SVM	6-degree-of-freedom knee kinematic data set	216
[51]	Nadimi-Shahraki et al.	2022	BE-WOA	KNN	The coronavirus 2019 dataset, 9 medical datasets form UCI and Kaggle	8–2000
[52]	Braik et al.	2023	ECSA, PCSA, SCSA	KNN	23 datasets from the UCI repository, KEEL repository, Kaggle, and another well-known medical website	9–5966
[53]	Singh et al.	2023	BFOAEPO	LR, RF, DT, KNN, SVM	Feature set derived from fundus images of the baseline dataset	36
[54]	Hashim et al.	2023	mHGS	KNN	19 UCI datasets represented by Parkinson’s disease phonation datasets	13–15009
[55]	Neggaz et al.	2024	MRFOSCA	KNN	20 UCI datasets	13–15009

To address these issues, in this study, the BIMGO algorithm was developed for feature selection for medical data, aiming to improve the robustness of the algorithm, the diversity of the solutions, and the balance of the search strategies. This algorithm was applied to the task of feature selection for medical data in multiple dimensions.

## III. Proposed algorithm

### A. Mountain gazelle optimizer

The MGO algorithm is a novel swarm intelligence algorithm [39], inspired by the social behavior and group life of mountain gazelles that live around the Arabian Peninsula. Mountain gazelles will form three groups in their life: female herds, single young male herds, and territorial male herds [56]. Every gazelle can become a member of the three groups during the optimization operation. Because young male gazelles are not mature, strong enough to mate or control the female herd, selecting a search population comprising one-third of the population in the MGO incur minimal costs.

The best global solution for the MGO in herd territories is adult male gazelles. The MGO uses four mechanisms for mathematical modeling, described as follows.

#### 1) Solitary territorial males

When male gazelles age and are sufficiently muscular, they choose areas far from other territories to establish and protect their own territory. Young male gazelles engage in fights when they attempt to challenge territorial males for territory or female gazelles. Adult territorial males are influenced by young males and the current optimal individual. The territory of an adult male is modeled as.(1):

$$TSM=male_{gazelle}-|(ri_{1}\times BH-ri_{2}\times X(t))\times F|\times Cof_{i}$$ (1)

where *male*_*gazelle*_ represents the location vector of the best individual. *ri*_1_ and *ri*_2_ are random numbers, with values of either 1 or 2. *BH* is the vector representing the effect factor of the minorities affected by search agents and nonsearch agents. *BH* is determined by Eq (2). The value of *F*, denoting the weights affected by the iteration, is calculated by Eq (3).


$$BH=X_{ra}\times \lfloor r_{1}\rfloor +M_{pr}\times \lceil r_{2}\rceil ,ra=\{\lceil \frac{N}{3}\rceil \cdots N\}$$ (2)

$$F=N_{1}(D)\times \exp(2-Iter\times (\frac{2}{MaxIter}))$$ (3)

In Eq (2), *ra* represents the interval of the presence of young individuals, and *X*_*ra*_ is the random solution within this interval, indicating young males. In the optimization process, *M*_*pr*_ is the average of $\left\lceil \frac{N}{3}\right\rceil$ randomly selected search agents. *r*_1_ and *r*_2_ are random values ranging from 0 to 1. *N* represents the population size. In Eq (3), *N*_1_ represents a random number from a standard distribution in the problem dimensions, exp is an exponential function, *Iter* denotes the iteration at present, and *MaxIter* indicates the maximum number of iterations.

To enhance the search capabilities, a coefficient vector *Cof*_*i*_ is proposed:

$$Cof_{i}=\{\begin{array}{c} (a+1)+r_{3},\\ a\times N_{2}(D),\\ r_{4}(D),\\ N_{3}(D)\times N_{4}{\left(D\right)}^{2}\times \cos(\left(r_{4}\times 2)\times N_{3}(D\right)) \end{array}$$ (4)

where *r*_3_ and *r*_4_ are random parameters, with values ranging from 0 to 1. *N*_2_, *N*_3_, and *N*_4_ are random numbers distributed within the dimensions of the problem, and *a* is a control parameter determined by Eq (5):

$$a=-1+Iter\times (\frac{-1}{MaxIter})$$ (5)

Eq (5) shows that *a* depends on the iteration process, and its value range is [−2,−1).

#### 2) Maternity herds

The continuation of the life cycle of the mountain gazelle group is inseparable from male gazelles reproducing with individuals in the female herd. Male gazelles play an important role when young males attempt to mate with females or when females give birth. Female herds are influenced by the best individual of the current iteration, randomized search agents, and young males; this behavior is expressed using Eq (6):

$$MH=(BH+Cof_{1,i})+(ri_{3}\times male_{gazelle}-ri_{4}\times X_{rand})\times Cof_{2,i}$$ (6)

where *BH* is calculated using Eq (2), *Cof*_1,*i*_ and *Cof*_2,*i*_ are random vectors calculated through Eq (4). *ri*_3_ and *ri*_4_ are random coefficients, each with a value of either 1 or 2. *X*_*rand*_ represents the vector position of a random individual.

#### 3) Bachelor male herds

When young male gazelles grow into adults, they establish their own territories and attempt to control female gazelles. During this process, young male individuals engage in violent fights with older males. This behavior can be expressed using Eq (7):

$$BMH=(X(t)-D)+(ri_{5}\times male_{gazelle}-ri_{6}\times BH)\times Cof_{i}$$ (7)

where *X*(*t*) is the location of the individual in the current iteration. *ri*_5_ and *ri*_6_ are random coefficients with values of either 1 or 2. *r*_6_ in Eq (8) is a random number ranging from 0 to 1. *D* denotes the vector of coefficients influenced by the current and optimal individuals, and its value is calculated by Eq (8):

$$D=(\left|X(t)|+|male_{gazelle}\right|)\times (2\times r_{6}-1)$$ (8)

#### 4) Migration to search for food

The maintenance of the life cycle of gazelles is inseparable from their food consumption. Gazelles continuously search for food and migrate, leveraging their impressive running and jumping abilities. This process can be expressed as Eq (9):

$$MSF=(ub-lb)\times r_{7}+lb$$ (9)

where *ub* and *lb* represent the upper and lower limits of the problem, respectively, and *r*_7_ is a random value between 0 and 1.

The above four mechanisms are utilized by all individuals to produce a new generation, which is then added to the population. This process of generating a new generation is akin to a duplicate. With the completion of each generation, the whole groups are arranged in ascending order. In general, the best individuals are protected in the group, while the poor individuals are removed.

### B. Improved mountain gazelle optimizer

To achieve high accuracy as well as fast convergence speed, four different mechanisms are adopted in the MGO. However, there are still some shortcomings, such as the imbalance between exploration and exploitation and the ability to easily fall into local optimal solutions. In an effort to improve the capability of the MGO and achieve better feature selection in medical data analysis, this paper presents an improved mountain gazelle optimizer. The IMGO method includes four innovations. First, ICMIC mapping is utilized as an initialization method, ensuring the multiplicity of the population and improving the early exploration capabilities of the algorithm. Second, the control factor *a* in the coefficient vector is replaced with a nonlinear factor that balances the global and local search capacities and improves the search efficiency. Third, a spiral perturbation mechanism is utilized to improve the local search capability. Eventually, the search is exploited in the optimal individual’s neighborhood to further enhance exploitation.

#### 1) Initialization of ICMIC mapping

In the MGO algorithm, the population is initialized randomly. This initialization strategy limits the algorithm’s performance because it does not ensure the diversity of solutions and may easily cause the algorithm to fall into local optima.

We use chaotic mapping as the initialization method to address this problem. The initial population obtained by this method covers the entire solution space, enhancing the global search capability. Additionally, the population created through chaotic mapping is uniformly distributed, aiding in minimizing the likelihood of becoming trapped in local optima. This approach contributes to the improvement of the convergence speed [57, 58]. Logistic mapping and tent mapping are the most common chaotic maps. However, these mapping approaches have a limited number of folds in their iterative regions. In contrast, the ICMIC [59] is an infinitely folded iterative chaotic map. Moreover, the ICMIC has high Lyapunov exponents; therefore, it has stronger chaotic properties than do other chaotic mappings. In addition, the ICMIC has the advantages of initial value sensitivity and uniform distribution. Therefore, in this paper, the ICMIC is used to initialize the population. The ergodicity of the ICMIC overcomes the shortcomings of traditional optimization algorithms by enabling better diversity in the initial state of the population, preventing premature convergence and improving the accuracy and convergence of global optimization.

Eq (10) provides the mathematical expression for the ICMIC:

$$X_{i+1}=ICMIC(X_{i})=sin(\frac{\alpha \pi }{X_{i}})$$ (10)

where *X*_*i*_∈[−1,0)∪(0,1] is the *i*-th gazelle individual and *α*∈(0,1) is the control parameter. A good chaotic sequence can only be obtained when α>0.6; hence, we set α = 0.7 and Eq (10) is then replaced by Eq (11):

$$X_{i+1}=ICMIC(X_{i})=sin(\frac{0.7\pi }{X_{i}})$$ (11)

Algorithm 1 shows the details of population initialization.

**Algorithm 1**. Population initialization using ICMIC mapping.

*ε* = 0.7*π*; % Setting parameter values

generate a random number *ω*_0_ within the range[−1.0)∪(0,1];

**for** each gazelle *i*from 1 to *N* **do**

  $\omega_{i}=ICMIC(\omega_{i-1})=sin(\frac{\varepsilon }{\omega_{i-1}})$; % Using ICMIC Chaos Mapping

  $X(i)=lb+\frac{\left(\omega_{i}+1\right)}{2}\times (ub-lb)$; % Updating the position after chaotic mapping

**end for**

#### 2) Nonlinear control factor

*Cof*_*r*_ is a coefficient vector randomly selected in each iteration, participating in the operation of three out of four mechanisms, namely TSM, MH, and BMH, and its value is largely determined by the control factor *a* in Eq (5).

**Fig 1:**
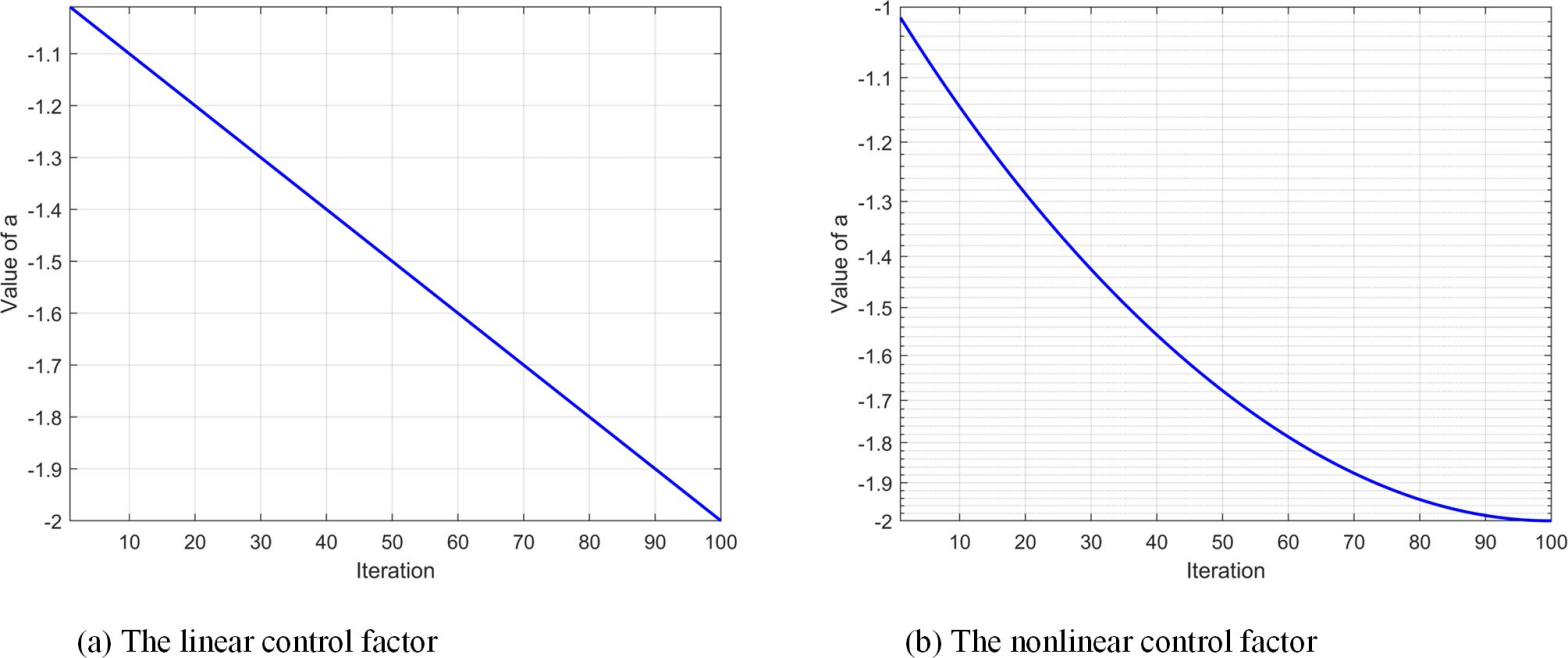
Iterative chart of control factor.

According to Eq (5), Fig 1(A) shows that the control factor linearly increases from -2 to -1. If the algorithm becomes stuck at a "nonideal point" in the initial phase, the constant change rate makes it prone to premature convergence to local optima. Therefore, we introduce a nonlinear control factor to improve the global search during the early stages, aiming for a comprehensive search for the solution domain. Additionally, the focus shifts to strengthening the local search in the later stages, seeking better possibilities within the known range. In this case, the control factor *a* is adjusted from Eq (5) to Eq (12).

$$a=-2+\log_{2}[1+{\left(1-\frac{t}{T}\right)}^{2}]$$ (12)

where *t* represents the current iteration, and *MaxIter* is the maximum number of iterations.

As shown in Fig 1(B), the preslope is large, and the nonlinear control factor changes quickly, which is more favorable than the linear control factor for increasing the search range and enhancing the global search ability at the initial iteration. When approaching *MaxIter*, the linear factor still maintains a uniform change. Thus, the search range cannot be effectively controlled, and the convergence ability of the algorithm is reduced, while the nonlinear control factor changes slowly, effectively controlling the search range, enhancing the local search ability, accelerating the convergence speed, and improving the quality of finding feasible solutions.

#### 3) Spiral perturbation mechanism

New individuals are continuously added during the iteration process through four mechanisms. However, there is no position update for the existing gazelles in the original algorithm, leading to insufficient local search capabilities and preventing the algorithm from converging quickly. Therefore, to increase the local search capacity, a spiral perturbation mechanism for gazelle individuals is introduced. The spiral search strategy is proposed in the WOA for modeling the behavior of whale populations in terms of rounding prey. During the iteration process, individual whales use this strategy to update their positions, thus increasing the diversity of individuals while ensuring the convergence speed of the algorithm.

Inspired by the spiral search in the whale optimization algorithm, the current individuals are perturbed after the gazelle population is updated; then, the fitness values of the new individuals obtained after the perturbation are compared with those before the perturbation, and the better individuals are retained in this paper. The perturbation process is influenced by the global optimal individual of the current iteration, and the perturbation method is a spiral search, which is shown in Eq (13):

$$X_{i}^{t+1}=male_{gazelle}+e^{c\times l}\times cos(2\pi l)\times |male_{gazelle}-X_{i}^{t}|$$ (13)

where *male*_*gazelle*_ is the optimal individual, *c* is the spiral shape constant, and *l* is the path coefficient, which is a random number within the range of [–1, 1].

By introducing the spiral perturbation mechanism and selecting the elite individuals among the individuals before and after the perturbation, the localized search capability of the current individual and the convergence speed can be effectively enhanced. This approach also augments the diversity of individuals and optimizes the overall search efficiency.

#### 4) Optimal individual neighborhood search

The gazelle algorithm utilizes four unique mechanisms named *TSM*, *MH*, *BMH*, and *MSF* for optimization, primarily focusing on strengthening its global search capabilities. However, its limited local search ability makes convergence challenging. To achieve a better equilibrium between exploration and exploitation throughout all optimization stages and given the high probability of the existence of globally optimal solutions within the optimal individuals and their neighborhoods, we introduced an optimal individual neighborhood search strategy.

This method records the optimal gazelle $male_{gazelle}^{t}$ and the suboptimal gazelle $male_{gazelle-1}^{t}$ in each iteration, defining an area between $male_{gazelle}^{t}$ and $male_{gazelle-1}^{t}$ as the optimal individual’s neighborhood. A local search is then conducted within this neighborhood, and the random individual *BN*^*t*^ within the neighborhood is mathematically represented by:

$$BN^{t}=male_{gazelle}^{t}+random(ub_{bn},lb_{bn})$$ (14)

where *random* represents a randomly selected gazelle within the neighborhood, *ub*_*bn*_ and *lb*_*bn*_ are the upper and lower bounds, respectively, and *t* is the current iteration number.

Finally, the fitness value of the individual obtained from Eq (14) is compared with that of the current best value to update the optimal individual *male*_*gazelle*_. Through the introduction of the optimal individual neighborhood search strategy, the local search of the neighborhood of the optimal and suboptimal individuals is strengthened, effectively improving the development and convergence abilities of the algorithm.

#### 5) Algorithm framework

**Fig 2:**
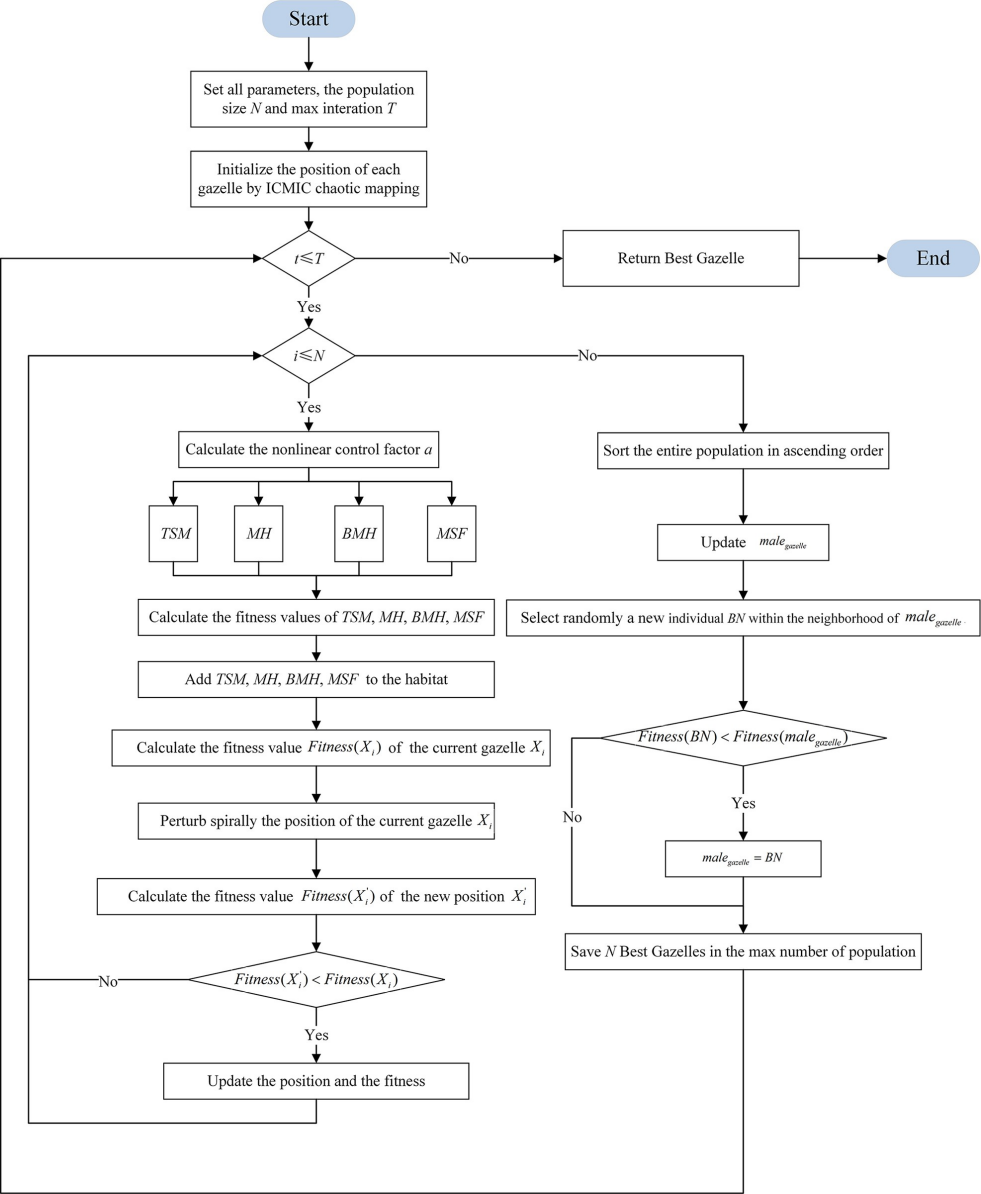
The flowchart of IMGO.

The process of the proposed IMGO is shown in Fig 2. Initially, the IMGO employs the ICMIC to initialize the gazelle population. In each iteration, nonlinear control factors are calculated. Subsequently, the IMGO updates the population in parallel using four distinct mechanisms. Then, a spiral perturbation is applied to adjust the positions of the gazelle individuals. Finally, the algorithm conducts a search in the optimal individual’s neighborhood to update the optimal individual. By improving the local exploitation capabilities and balancing global and local searches, the algorithm effectively enhances the convergence speed and overall performance. The pseudocode for the IMGO is detailed in Algorithm 2.

**Algorithm 2**. Pseudocode of IMGO.

1: Initialize Population size *N*, maximum iterations *T*;

2: Set all parameters;

3: Initialize a population ${\left\{Xi\right\}}_{i=1}^{N}$ based on Algorithm 1; %Initialization of populations using ICMIC

4: Calculate fitness levels of gazelles;

5: **While** (current iteration *t*<*T* ) **do**

6: **For** each gazelle *i* from 1 to *N* **do**

7: Calculate nonlinear control factor *a* using Eq (12); %Introducing a nonlinear control factor

8: Calculate *TSM* with Eq (1);

9: Calculate *MH* with Eq (6);

10: Calculate *BMH* with Eq (7);

11: Calculate *MSF* with Eq (9);

12: Calculate the fitness values of *TSM*, *MH*, *BMH*, and *MSF*, add them to the habitat;

13: % spiral perturbation mechanism

14: Spirally perturb the current gazelle’s position based on Eq (13); % Perturbing the current individual using the spiral perturbation mechanism

15: **if** fitness value of the new location < fitness value of the original location **then** %Selecting the elite among current individuals and new individuals after perturbation

16: Update location;

17: Update fitness value;

18: **end if**

19: **end for**

20: Sort all individuals of the population in ascending order;

21: Update *best*_*Gazelle*_;

22: % The search in optimal individual’s neighborhood 

23: Generate a new individual Y randomly in the $\left[best_{Gazelle}-abs(best_{Gazelle}-sbest_{Gazelle}),best_{Gazelle}+abs(best_{Gazelle}-sbest_{Gazelle})\right]$;

24: **If** the fitness value of *Y* < the fitness value of *best*_*Gazelle*_ **then** % Selecting of the current best individual and the better individual in its neighborhood

25: *best*_*Gazelle*_ = *Y*;

26: **end if**

27: Retain the *N* best individuals; % Weeding out bad individuals

28: **end while**

29: Returns the best fitness value of *X*_*BestGazelle*_;

### C. Binary improved mountain gazelle optimizer

The IMGO algorithm is suitable for continuous search spaces, but for discrete search spaces, the algorithm cannot be applied directly. To this end, we developed a binary version of the IMGO, named the BIMGO. In an effort to address discrete optimization issues, a sigmoid function that maps continuous space into discrete space is utilized. Eq (15) was utilized to develop the binary enhanced mountain gazelle algorithm:

$$S(X_{i})=\frac{1}{1+e^{-X_{i}}}$$ (15)

where *S*(*X*_*i*_) represents the probability of changing its binary position. Then, a threshold needs to be set. To ensure a uniform distribution of 0 to 1 in the discrete space, we set the threshold to 0.5. The update of position *B*_*i*_ is given by Eq (16):

$$B_{i}=\{\begin{array}{l} 0\;if\;S(X_{i})<0.5\\ 1\;if\;S(X_{i})\geq 0.5 \end{array}$$ (16)

Algorithm 3 represents the optimized process of the binary conversion process by using pseudocode.

Algorithm 3. Pseudocode of binary conversion process.

1: **Input** the population ${\left\{Xi\right\}}_{i=1}^{N}$;

2: **Initialize** threshold *r* = 0.5;

3: **for** each gazelle *i* from 1 to *N* **do**

4: **Calculate** *S*(*Xi*) based on Eq (15);

5: **if *S*(*Xi*)≥** *r* **then**

6: *B*_*i*_ = 1;

7: **else**

8: *B*_*i*_ = 0;

9: **end if**

10: **end for**

11: **Return *B***_***i***_;

## IV. Experiments and results

### A. Experiment on benchmark functions

To validate the effectiveness and performance of the proposed IMGO algorithm, we compared the IMGO algorithm and the original MGO algorithm together with eight well-known optimization algorithms on 23 benchmark functions. The eight algorithms are the WOA, PSO, GWO, SSA, MPA, NOA, KOA, and BA.

#### 1) Parameter settings and benchmark functions

Table 2 shows the settings of the relevant parameters used in the experiment. The parameters of all comparative algorithms are set on the basis of their original papers.

**Table 2:** Parameter settings of the comparison algorithms.

Algorithm	Parameter	Value
BA	*Qmin*	0
	*Qmax*	2
GWO	*a*	[2,0]
KOA	Constant	15
	Initial gravitational value	0.1
	Control parameter	3
NOA	*δ*	0.05
	*P*_*a*1_	0.2
	*P*_*a*2_	0.4
PSO	*ω*	[0.2,0.9]
	c_1_	2
	*c*_2_	2
SSA	Leader position update probability	0.5
WOA	*a*	[2,0]
	*b*	1

All experiments were performed under the same conditions. The computer used for these experiments was equipped with an Intel Core i7, 2.8 GHz CPU, and 16 GB of RAM. All experiments were implemented in MATLAB 2023a and run on the same computer with the Windows 10 operating system.

The representation of the IMGO and comparative algorithms is evaluated using 23 fundamental benchmark functions given in Table 3. The functions are primarily separated into 3 categories: unimodal benchmark functions (*f*1−*f*7), multimodal benchmark functions (*f*8−*f*13), and fixed-dimensional multimodal benchmark functions (*f*14−*f*23).

**Table 3:** Details of 23 basic benchmark functions.

	Function	Dim.	Range	Optimal value
**unimodal benchmark functions**	$f_{1}(x)=\sum_{i=1}^{Dim}x_{i}^{2}$	30	[−100,100]^*Dim*^	0
	$f_{2}(x)=\sum_{i=1}^{Dim}\left|x_{i}\right|+\prod_{i=1}^{Dim}\left|x_{i}\right|$	30	[−10,10]^*Dim*^	0
	$f_{3}(x)=\sum_{i=1}^{Dim}{\left(\sum_{j=1}^{i}x_{j}\right)}^{2}$	30	[−100,100]^*Dim*^	0
	$f_{4}(x)=max_{i}\{|x_{i}|,1\leq i\leq Dim\}$	30	[−100,100]^*Dim*^	0
	$f_{5}(x)=\sum_{i=1}^{Dim-1}\left[100{\left(x_{i+1}-x_{i}^{2}\right)}^{2}+{\left(x_{i}-1\right)}^{2}\right]$	30	[−30,30]^*Dim*^	0
	$f_{6}(x)=\sum_{i=1}^{Dim}{\left(x_{i}+0.5\right)}^{2}$	30	[−100,100]^*Dim*^	0
	$f_{7}(x)=\sum_{i=1}^{Dim}ix_{i}^{4}+random[0,1)$	30	[−1.28,1.2]^*Dim*^	0
**multimodal benchmark functions**	$f_{8}(x)=\sum_{i=1}^{Dim}-x_{i}sin(\sqrt{\left|x_{i}\right|})$	30	[−500,500]^*Dim*^	−418.9829×*Dim*
	$f_{9}(x)=\sum_{i=1}^{Dim}\left[x_{i}^{2}-10cos(2\pi x_{i})+10\right]$	30	[−5.12,5.12]^*Dim*^	0
	$f_{10}(x)=-20exp(-0.2\sqrt{\frac{\sum_{i=1}^{Dim}x_{i}^{2}}{Dim}})-exp(\frac{\sum_{i=1}^{Dim}cos(2\pi x_{i})}{Dim})+20+e$	30	[−32,32]^*Dim*^	0
	$f_{11}(x)=\frac{1}{4000}\sum_{i=1}^{Dim}x_{i}^{2}-\prod_{i=1}^{Dim}cos(\frac{x_{i}}{\sqrt{i}})+1$	30	[−600,600]^*Dim*^	0
	$\begin{array}{l} f_{12}(x)=\frac{\pi }{n}\{10sin(\pi y_{i})+\sum_{i=1}^{Dim-1}{\left(y_{i}-1\right)}^{2}[1+10sin^{2}(\pi y_{i+1})]\\ -{\left(y_{Dim}-1\right)}^{2}\}+\sum_{i=1}^{Dim}u(x_{i},5,100,4) \end{array}$ $\begin{array}{l} y_{i}=1+\frac{1}{4}(x_{i}+1)\\ u(x_{i},a,k,m)=\left\{ \begin{array}{c} k{\left(x_{i}-a\right)}^{m},x_{i}>a\\ 0,-a<x_{i}<a\\ k{\left(-x_{i}-a\right)}^{m},x_{i}<-a \end{array}\right. \end{array}$	30	[−50,50]^*Dim*^	0
	$\begin{array}{l} f_{13}(x)=0.1\{sin^{2}(3\pi x_{i})+\sum_{i=1}^{Dim}{\left(x_{i}-1\right)}^{2}[1+sin^{2}(3\pi x_{i}+1)]\\ +{\left(x_{Dim}-1\right)}^{2}[1+sin^{2}(2\pi x_{Dim})]\}+\sum_{i=1}^{Dim}u(x_{i},5,100,4) \end{array}$	30	[−50,50]^*Dim*^	0
**fixed-dimension multimodal benchmark functions.**	$f_{14}(x)={\left[\frac{1}{500}+\sum_{j=1}^{25}\frac{1}{j+\sum_{i=1}^{2}{\left(x_{i}-a_{ij}\right)}^{6}}\right]}^{-1}$	2	[−65,65]^*Dim*^	1
	$f_{15}(x)=\sum_{i=1}^{11}{\left[a_{i}-\frac{\left[x_{1}(b_{i}^{2}+b_{i}x_{2})\right]}{b_{i}^{2}+b_{i}x_{3}+x_{4}}\right]}^{2}$	4	[−5,5]^*Dim*^	0.00030
	$f_{16}(x)=4x_{1}^{2}-2.1x_{1}^{4}+\frac{1}{3}x_{1}^{6}+x_{1}x_{2}-4x_{2}^{2}+4x_{2}^{4}$	2	[−5,5]^*Dim*^	-1.0316
	$f_{17}(x)={\left(x_{2}-\frac{5.1}{4\pi^{2}}x_{1}^{2}+\frac{5}{\pi }x_{1}-6\right)}^{2}+10(1-\frac{1}{8\pi })cosx_{1}+10$	2	[−5,5]^*Dim*^	0.398
	$\begin{array}{l} f_{18}(x)=[1+{\left(x_{1}+x_{2}+1\right)}^{2}(19-14x_{1}+3x_{1}^{2}-14x_{2}+6x_{1}x_{2}\\ +3x_{2}^{2})]\times {[30+{\left(2x_{1}-3x_{2}\right)}^{2}(18-32x_{1}+12x_{1}^{2}+48x}_{2}-36x_{1}x_{2}\\ +27x_{2}^{2})] \end{array}$	2	[−2,2]^*Dim*^	3
	$f_{19}(x)=-\sum_{i=1}^{4}c_{i}\exp[-\sum_{j=1}^{3}a_{ij}{\left(x_{i}-p_{ij}\right)}^{2}]$	3	[1,3]^*Dim*^	-3.86
	$f_{20}(x)=-\sum_{i=1}^{4}c_{i}\exp[-\sum_{j=1}^{6}a_{ij}{\left(x_{i}-p_{ij}\right)}^{2}]$	6	[0,1]^*Dim*^	-3.32
	$f_{21}(x)=-\sum_{i=1}^{5}{\left[(X-a_{i}){\left(X-a_{i}\right)}^{T}+c_{i}\right]}^{-1}$	4	[0,10]^*Dim*^	-10.1532
	$f_{22}(x)=-\sum_{i=1}^{7}{\left[(X-a_{i}){\left(X-a_{i}\right)}^{T}+c_{i}\right]}^{-1}$	4	[0,10]^*Dim*^	-10.4028
	$f_{23}(x)=-\sum_{i=1}^{10}{\left[(X-a_{i}){\left(X-a_{i}\right)}^{T}+c_{i}\right]}^{-1}$	4	[0,10]^*Dim*^	-10.5363

When evaluating the algorithm on benchmark functions, the population size was set to 30, the number of independent runs was set to 20, and the maximum iterative number was set to 500.

#### 2) Experimental results and discussion

The optimal values, mean values, and standard deviations are adopted for comparative analysis. The optimal algorithm is determined based on the mean value, which is highlighted in bold. In cases where algorithms share identical mean values for a function, the standard deviation, which is regarded as superior when it is smaller, is then considered a deciding factor. Moreover, a 5% Wilcoxon rank-sum test is used to determine whether significant differences exist in the performance between the IMGO algorithm and competing algorithms on various functions.

According to the principles of the ’No Free Lunch (NFL)’ theorem [51, 60], no algorithm can achieve optimal results across all test functions. In addition to evaluating the performance of the IMGO algorithm against the other eight algorithms, it is also important to compare the IMGO algorithm with the original MGO algorithm because the IMGO algorithm is an improved version of the MGO algorithm. Therefore, in cases where the IMGO exhibits better performance than that of the MGO but does not rank as the best overall, the results are underlined.

The results of the experiments between the IMGO and its comparative algorithms on basic unimodal benchmark functions are presented in Table 4.

**Table 4:** Experimental results on basic unimodal benchmark functions (*f*1−*f*7).

Function		BA	GWO	PSO	SSA	WOA	KOA	NOA	MPA	MGO	IMGO
***f*1**	Mean	1.92E+04	1.78E-27	2.51E-06	2.89E-07	5.05E-74	1.38E+04	3.60E-12	8.48E-23	7.91E-74	**2.40E-84**
	Std	8.17E+03	1.91E-27	1.05E-05	4.64E-07	1.75E-73	2.82E+03	8.85E-12	1.54E-22	2.36E-73	**1.06E-83**
	Best	1.01E+04	2.98E-29	1.84E-13	3.39E-08	5.67E-84	8.72E+03	1.61E-77	2.20E-24	8.44E-80	**4.16E-93**
	Rank	10	4	8	7	2	9	6	5	3	1
***f*2**	Mean	6.09E+06	8.48E-17	6.47E-02	2.15E+00	**5.57E-52**	4.02E+02	4.54E+00	2.94E-13	1.62E-41	2.99E-46
	Std	2.72E+07	6.68E-17	9.57E-02	1.54E+00	**1.48E-51**	1.20E+03	2.03E+01	2.97E-13	3.97E-41	7.37E-46
	Best	4.98E+01	1.13E-17	3.16E-03	1.85E-01	**2.81E-57**	4.25E+01	1.22E-42	2.16E-14	3.48E-46	3.22E-50
	Rank	10	4	6	7	1	9	8	5	3	2
***f*3**	Mean	5.67E+04	1.60E-05	7.95E+01	1.40E+03	4.37E+04	4.16E+04	1.34E-03	5.79E-05	6.54E-08	**1.03E-09**
	Std	2.43E+04	4.94E-05	7.94E+01	7.18E+02	1.77E+04	1.44E+04	6.01E-03	5.46E-05	2.57E-07	**3.79E-09**
	Best	1.70E+04	2.54E-08	1.61E+01	3.81E+02	1.86E+04	2.28E+04	1.15E-84	1.81E-07	3.04E-17	**9.27E-21**
	Rank	10	3	6	7	9	8	5	4	2	1
***f*4**	Mean	5.43E+01	8.91E-07	2.05E+00	1.09E+01	5.58E+01	5.44E+01	2.35E-01	2.97E-09	2.12E-24	**1.02E-35**
	Std	9.73E+00	1.01E-06	9.12E-01	3.76E+00	2.74E+01	5.74E+00	1.05E+00	1.18E-09	5.21E-24	**3.82E-35**
	Best	3.45E+01	1.09E-07	7.48E-01	3.79E+00	7.94E+00	4.56E+01	4.99E-32	1.02E-09	7.35E-31	**5.97E-41**
	Rank	8	4	6	7	10	9	5	3	2	1
***f*5**	Mean	2.64E+07	2.70E+01	5.11E+01	2.83E+02	2.80E+01	1.48E+07	2.90E+01	2.52E+01	2.22E-22	**6.16E-32**
	Std	1.76E+07	5.93E-01	3.38E+01	4.18E+02	4.56E-01	6.42E+06	3.58E-02	3.30E-01	9.91E-22	**2.76E-31**
	Best	7.39E+06	2.61E+01	8.60E+00	2.70E+01	2.71E+01	5.28E+06	2.88E+01	2.48E+01	0.00E+00	**0.00E+00**
	Rank	10	4	7	8	5	9	6	3	2	1
***f*6**	Mean	2.13E+04	8.09E-01	2.18E-06	1.52E-07	3.57E-01	1.28E+04	8.68E+02	3.84E-08	**7.80E-10**	7.34E-07
	Std	8.11E+03	4.60E-01	6.51E-06	1.41E-07	2.01E-01	2.90E+03	3.85E+03	2.11E-08	**1.23E-09**	9.89E-07
	Best	1.14E+04	1.11E-01	2.60E-13	2.75E-08	1.22E-01	7.89E+03	4.46E+00	1.42E-08	**7.84E-14**	1.00E-09
	Rank	10	7	5	3	6	9	8	2	1	4
***f*7**	Mean	1.02E+01	2.34E-03	2.71E-02	2.01E-01	3.57E-03	8.29E+00	2.39E-02	1.96E-03	5.30E-04	**1.79E-04**
	Std	4.83E+00	1.19E-03	1.13E-02	8.12E-02	4.41E-03	3.20E+00	2.47E-02	9.07E-04	3.31E-04	**1.25E-04**
	Best	2.18E+00	5.15E-04	1.08E-02	8.48E-02	2.20E-04	2.94E+00	4.72E-04	7.63E-04	7.27E-05	**1.56E-06**
	Rank	10	4	7	8	5	9	6	3	2	1
Average rank		9.7	4.3	6.4	6.7	5.4	8.9	6.3	3.6	2.1	1.6

For *f*1, *f*3, *f*4, *f*5, and *f*7, the IMGO exhibited superior performance with respect to the mean values and standard deviations. For function *f*2, the IMGO was second-best algorithm, ranking behind the WOA, its performance surpassed that of the original MGO. In the evaluation of function *f*6, the IMGO was fourth-best algorithm, and ranking behind the MGO among all the compared algorithms. The average ranking of IMGO’s performance on function *f*1−*f*7 is 1.6, which is the best among all algorithms.

**Fig 3:**
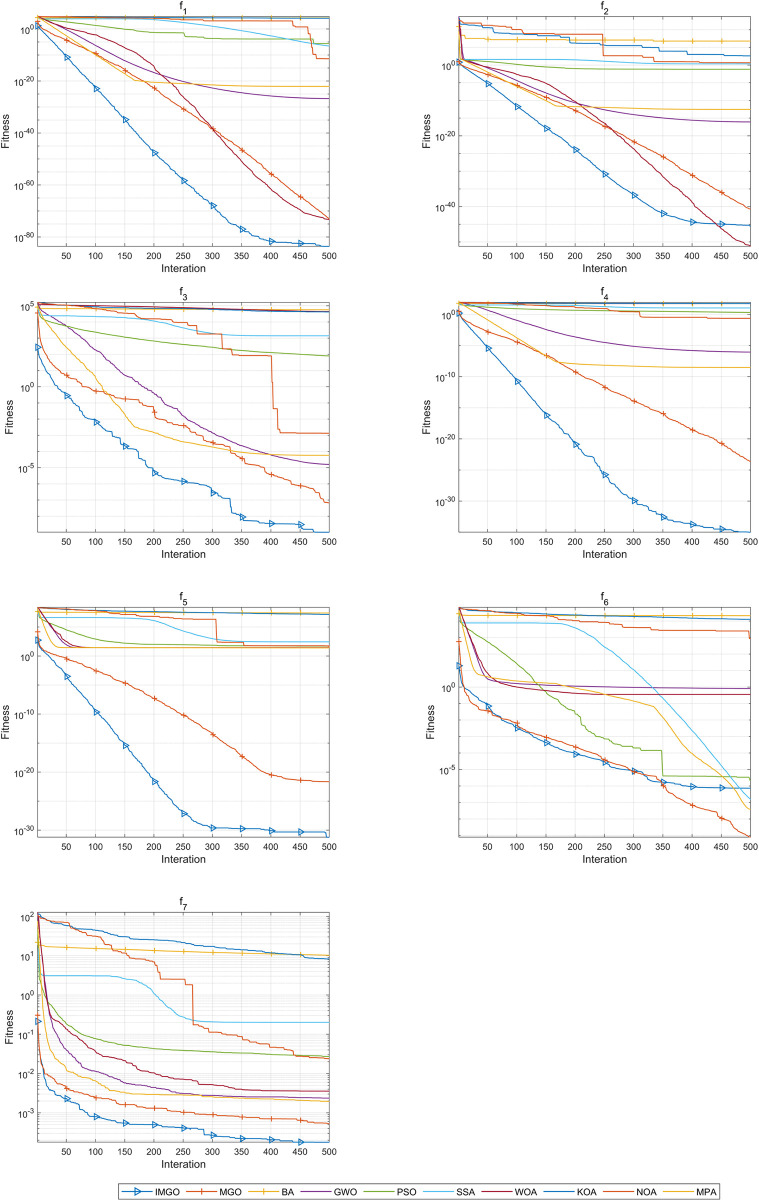
Convergence curves of the IMGO and comparison algorithms on functions *f*1−*f*7.

Fig 3 shows the convergence curves of each method on different functions. It is observed that the IMGO algorithm converged best on functions *f*1, *f*3, *f*4, *f*5, and *f*7. In particular, the convergence ability of the IMGO was the most pronounced on functions *f*1, *f*4, and *f*5, where it rapidly converged to the optimal solution. The proposed IMGO algorithm exhibited the weakest performance on function *f*6; however, it ranked fourth in terms of overall convergence. Although the proposed algorithm ranked second on function *f*2, its overall convergence efficiency surpassed that of the original MGO. The IMGO algorithm exhibited excellent search capability and converged to the extremes very quickly throughout the iterations.

Based on the above analysis, the IMGO performed best on functions *f1-f7*, reflecting the ability of this algorithm to handle simple challenges.

In Table 5, the results of the tests on multimodal benchmark functions are presented.

**Table 5:** Experimental results on basic multimodal benchmark functions (*f*8−*f*13).

Function		BA	GWO	PSO	SSA	WOA	KOA	NOA	MPA	MGO	IMGO
***f*8**	Mean	-1.77E+271	-5.90E+03	-6.63E+03	-7.47E+03	-9.71E+03	-3.86E+03	-4.51E+03	-8.93E+03	-1.26E+04	**-1.26E+04**
	Std	NaN	1.09E+03	5.64E+02	6.67E+02	1.56E+03	4.24E+02	8.61E+02	5.29E+02	7.52E-08	**2.28E-06**
	Best	-2.98E+272	-7.04E+03	-7.63E+03	-8.76E+03	-1.23E+04	-4.56E+03	-6.27E+03	-9.72E+03	-1.26E+04	**-1.26E+04**
	Rank	10	7	6	5	3	9	8	4	2	1
***f*9**	Mean	9.55E+01	3.34E+00	5.21E+01	5.11E+01	5.68E-15	2.87E+02	3.62E-01	**0.00E+00**	**0.00E+00**	**0.00E+00**
	Std	3.62E+01	3.65E+00	1.38E+01	1.68E+01	2.54E-14	2.64E+01	1.62E+00	**0.00E+00**	**0.00E+00**	**0.00E+00**
	Best	5.77E+01	5.68E-14	2.89E+01	2.39E+01	0.00E+00	2.26E+02	0.00E+00	**0.00E+00**	**0.00E+00**	**0.00E+00**
	Rank	9	6	8	7	4	10	5	1	1	1
***f*10**	Mean	1.64E+01	1.00E-13	1.18E+00	2.78E+00	4.35E-15	1.91E+01	1.53E-03	1.58E-12	1.15E-15	**7.99E-16**
	Std	1.05E+00	1.17E-14	8.35E-01	8.88E-01	2.55E-15	8.32E-01	6.82E-03	8.94E-13	1.46E-15	**1.09E-15**
	Best	1.38E+01	7.86E-14	2.27E-07	1.34E+00	4.44E-16	1.72E+01	4.44E-16	4.23E-13	4.44E-16	**4.44E-16**
	Rank	9	4	7	8	3	10	6	5	2	1
***f*11**	Mean	1.74E+02	1.38E-03	4.45E-02	1.97E-02	5.13E-03	1.32E+02	6.13E-02	**0.00E+00**	**0.00E+00**	**0.00E+00**
	Std	5.04E+01	4.40E-03	6.29E-02	1.32E-02	2.29E-02	2.81E+01	2.73E-01	**0.00E+00**	**0.00E+00**	**0.00E+00**
	Best	9.44E+01	0.00E+00	1.73E-11	7.46E-04	0.00E+00	7.69E+01	0.00E+00	**0.00E+00**	**0.00E+00**	**0.00E+00**
	Rank	10	4	7	6	5	9	8	1	1	1
***f*12**	Mean	1.54E+07	4.48E-02	9.85E-02	6.81E+00	2.19E-02	1.54E+07	1.07E+00	4.31E-06	**5.89E-26**	3.18E-20
	Std	1.38E+07	2.20E-02	1.48E-01	2.98E+00	1.21E-02	1.15E+07	3.67E-01	1.56E-05	**1.31E-25**	6.42E-20
	Best	1.31E+06	1.30E-02	1.20E-12	2.04E+00	1.94E-03	2.28E+06	5.59E-01	2.87E-09	**2.94E-29**	2.16E-24
	Rank	10	5	6	8	4	9	7	3	1	2
***f*13**	Mean	7.27E+07	5.54E-01	5.20E-02	1.98E+01	5.77E-01	4.77E+07	2.79E+00	1.38E-02	1.69E-32	**1.35E-32**
	Std	8.57E+07	2.54E-01	9.18E-02	1.40E+01	3.29E-01	2.93E+07	2.42E-01	2.49E-02	5.56E-33	**2.81E-48**
	Best	1.84E+07	8.59E-02	5.95E-12	5.29E-03	1.75E-01	1.46E+07	2.17E+00	5.82E-08	1.35E-32	**1.35E-32**
	Rank	10	5	4	8	6	9	7	3	2	1
Average rank		9.7	5.2	6.3	7.0	4.2	9.3	6.8	2.8	1.5	1.2

The IMGO algorithm attained the best mean among all algorithms on functions *f*8, *f*9, *f*10, *f*11, and *f*13. For functions *f*9 and *f*11, the MGO and IMGO achieved the best results in terms of the optimal solution, mean, and standard deviation. On function *f*12, the capacity of IMGO was only slightly worse than that of the MGO. The average ranking of IMGO’s performance on function *f*8−*f*13 is 1.2, which is the best among all algorithms.

**Fig 4:**
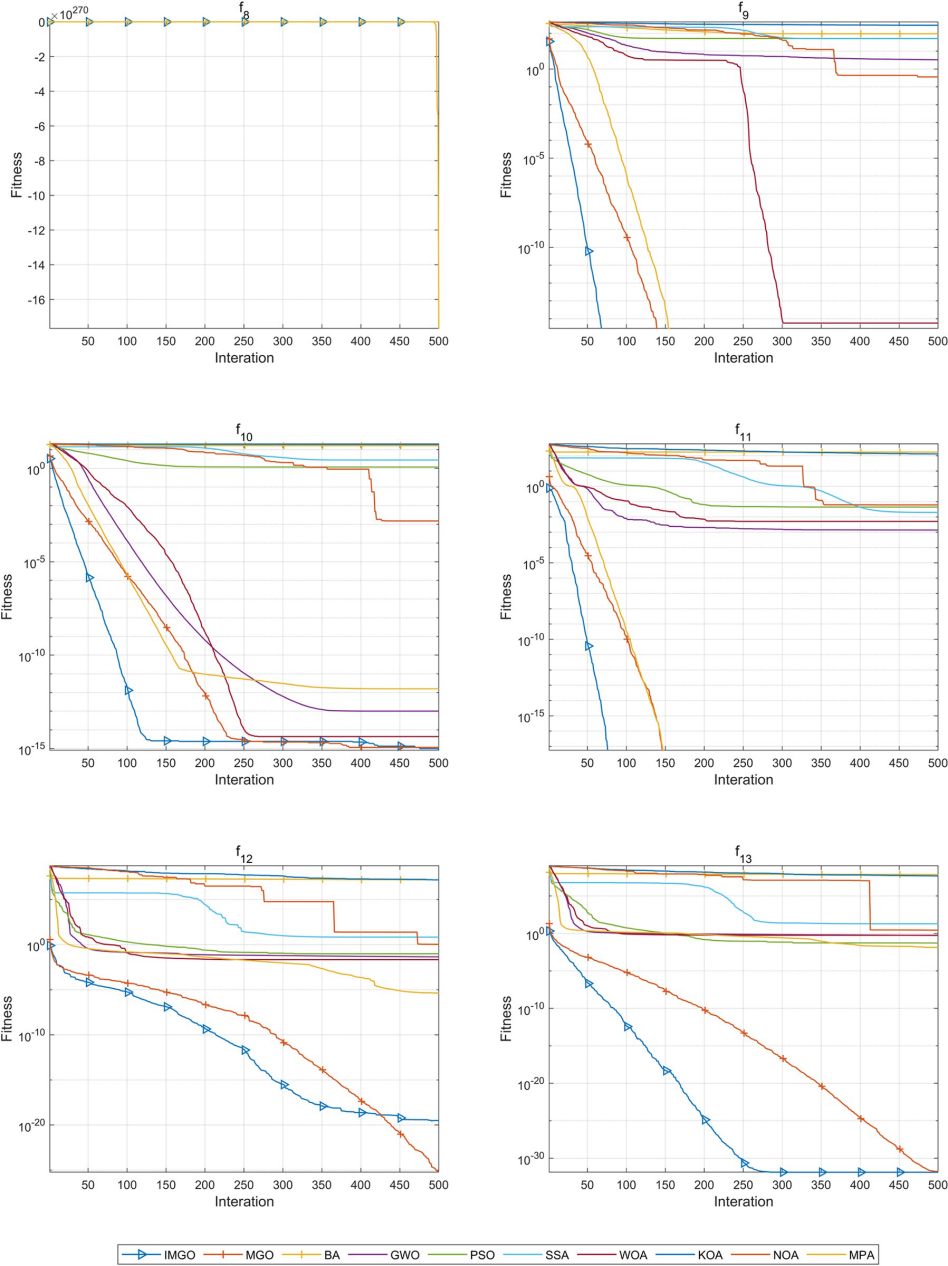
Convergence curves of the IMGO algorithm and comparison algorithms on functions *f*8−*f*13.

Fig 4 shows the convergence curves, highlighting that the IMGO exhibits the best convergence performance on functions *f*8, *f*9, *f*10, *f*11, and *f*13. Notably, this algorithm converges to the optimum in fewer than 100 iterations on both *f*9 and *f*11. The IMGO algorithm also shows strong convergence on *f*13, converging to the optimal solution at 300 iterations. Although the performance of the MGO surpassed that of the IMGO on function *f*12, the IMGO significantly outperformed the other algorithms. By synthesizing the data from Table 5 and Fig 4, the conclusion can be drawn that the IMGO exhibits the best performance on functions *f*8−*f*13.

Table 6 presents the performance of the IMGO algorithm in comparison to that of other methods on fixed-dimensional multimodal benchmark functions. The IMGO algorithm achieved the best means and standard deviation on function *f*18, *f*19, *f*21, *f*22, and *f*23.Although IMGO also has the best mean on function *f*14, *f*16, and *f*17, its standard deviation is not as good as that of MPA on function *f*14, and not as good as that of MPA and MGO on function *f*16 and *f*17.

**Table 6:** Experimental results on basic fixed-dimensional multimodal benchmark functions (*f*14−*f*23).

Function		BA	GWO	PSO	SSA	WOA	KOA	NOA	MPA	MGO	IMGO
***f*14**	Mean	9.32E+00	3.36E+00	4.24E+00	1.05E+00	2.23E+00	3.74E+00	6.35E+00	**9.98E-01**	9.98E-01	9.98E-01
	Std	7.25E+00	3.31E+00	3.67E+00	2.22E-01	2.22E+00	2.24E+00	3.79E+00	**8.82E-17**	2.55E-16	2.39E-16
	Best	9.98E-01	9.98E-01	9.98E-01	9.98E-01	9.98E-01	1.00E+00	1.13E+00	**9.98E-01**	9.98E-01	9.98E-01
	Rank	10	6	8	4	5	7	9	1	1	1
***f*15**	Mean	1.62E-02	3.51E-03	1.38E-03	2.87E-03	6.90E-04	6.81E-03	7.49E-03	**3.07E-04**	3.55E-04	3.08E-04
	Std	3.35E-02	7.27E-03	4.47E-03	5.99E-03	5.08E-04	5.72E-03	7.52E-03	**3.56E-15**	2.04E-04	5.98E-08
	Best	4.24E-04	3.09E-04	3.07E-04	5.49E-04	3.15E-04	1.58E-03	1.04E-03	**3.07E-04**	3.07E-04	3.07E-04
	Rank	10	7	5	6	4	8	9	1	3	2
***f*16**	Mean	-9.91E-01	-1.03E+00	-1.03E+00	-1.03E+00	-1.03E+00	-9.85E-01	-1.02E+00	**-1.03E+00**	-1.03E+00	-1.03E+00
	Std	1.82E-01	1.47E-08	2.10E-16	4.93E-14	4.81E-09	5.43E-02	9.30E-03	**0.00E+00**	1.44E-16	1.97E-16
	Best	-1.03E+00	-1.03E+00	-1.03E+00	-1.03E+00	-1.03E+00	-1.03E+00	-1.03E+00	**-1.03E+00**	-1.03E+00	-1.03E+00
	Rank	9	7	1	5	6	10	8	1	1	1
***f*17**	Mean	3.98E-01	3.98E-01	3.98E-01	3.98E-01	3.98E-01	4.26E-01	4.26E-01	**3.98E-01**	**3.98E-01**	3.98E-01
	Std	5.20E-10	1.10E-06	0.00E+00	6.73E-15	1.09E-05	2.80E-02	2.98E-02	**0.00E+00**	**0.00E+00**	3.98E-16
	Best	3.98E-01	3.98E-01	3.98E-01	3.98E-01	3.98E-01	3.98E-01	3.98E-01	**3.98E-01**	**3.98E-01**	3.98E-01
	Rank	6	7	1	5	8	10	9	1	1	1
***f*18**	Mean	5.70E+00	3.00E+00	3.00E+00	3.00E+00	3.00E+00	3.80E+00	5.80E+00	3.00E+00	3.00E+00	**3.00E+00**
	Std	8.31E+00	4.14E-05	1.08E-15	1.62E-13	9.34E-05	8.57E-01	6.13E+00	1.34E-15	2.00E-15	**5.05E-15**
	Best	3.00E+00	3.00E+00	3.00E+00	3.00E+00	3.00E+00	3.01E+00	3.01E+00	3.00E+00	3.00E+00	**3.00E+00**
	Rank	9	6	2	5	7	8	10	2	2	1
***f*19**	Mean	-3.86E+00	-3.86E+00	-3.86E+00	-3.86E+00	-3.85E+00	-3.84E+00	-3.85E+00	-3.86E+00	-3.86E+00	**-3.86E+00**
	Std	5.47E-08	2.81E-03	2.28E-15	5.00E-12	1.62E-02	1.82E-02	1.47E-02	2.12E-15	1.76E-15	**2.09E-15**
	Best	-3.86E+00	-3.86E+00	-3.86E+00	-3.86E+00	-3.86E+00	-3.86E+00	-3.86E+00	-3.86E+00	-3.86E+00	**-3.86E+00**
	Rank	6	7	1	5	8	10	9	1	1	1
***f*20**	Mean	-3.26E+00	-3.24E+00	-3.28E+00	-3.24E+00	-3.24E+00	-2.87E+00	-2.94E+00	**-3.32E+00**	-3.24E+00	-3.25E+00
	Std	6.07E-02	7.82E-02	5.82E-02	6.85E-02	9.79E-02	1.92E-01	1.52E-01	**1.40E-11**	5.82E-02	5.98E-02
	Best	-3.32E+00	-3.32E+00	-3.32E+00	-3.32E+00	-3.32E+00	-3.15E+00	-3.24E+00	**-3.32E+00**	-3.32E+00	-3.32E+00
	Rank	3	6	2	7	8	10	9	1	5	4
***f*21**	Mean	-4.27E+00	-9.64E+00	-6.03E+00	-7.76E+00	-8.74E+00	-1.97E+00	-3.67E+00	-1.02E+01	-1.02E+01	**-1.02E+01**
	Std	2.32E+00	1.56E+00	3.83E+00	3.10E+00	2.52E+00	1.02E+00	1.55E+00	2.71E-11	2.08E-15	**1.41E-15**
	Best	-1.02E+01	-1.02E+01	-1.02E+01	-1.02E+01	-1.02E+01	-5.18E+00	-7.45E+00	-1.02E+01	-1.02E+01	**-1.02E+01**
	Rank	8	4	7	6	5	10	9	3	1	1
***f*22**	Mean	-4.43E+00	-1.04E+01	-7.13E+00	-8.34E+00	-8.25E+00	-2.48E+00	-3.51E+00	-1.04E+01	-1.04E+01	**-1.04E+01**
	Std	2.79E+00	9.43E-04	3.75E+00	3.29E+00	2.67E+00	1.42E+00	1.39E+00	6.14E-11	3.69E-15	**2.16E-15**
	Best	-1.04E+01	-1.04E+01	-1.04E+01	-1.04E+01	-1.04E+01	-8.19E+00	-7.22E+00	-1.04E+01	-1.04E+01	**-1.04E+01**
	Rank	8	4	7	5	6	10	9	3	1	1
***f*23**	Mean	-4.61E+00	-9.72E+00	-7.95E+00	-8.71E+00	-8.17E+00	-2.41E+00	-3.83E+00	-1.05E+01	-1.05E+01	**-1.05E+01**
	Std	3.16E+00	2.50E+00	3.67E+00	3.28E+00	3.03E+00	1.01E+00	1.32E+00	6.69E-11	2.97E-15	**1.68E-15**
	Best	-1.05E+01	-1.05E+01	-1.05E+01	-1.05E+01	-1.05E+01	-4.94E+00	-6.53E+00	-1.05E+01	-1.05E+01	**-1.05E+01**
	Rank	8	4	7	5	6	10	9	3	1	1
Average rank		7.7	5.8	4.1	5.3	6.3	9.3	9.0	1.7	1.7	1.4

IMGO performs only slightly worse than MPA on function *f*15, but better than MGO. IMGO performs average on function *f*20, only ranking fourth, but better than MGO. In the table, we used the Scientific notation, which led to the fact that the values were not fully displayed. Therefore, although some values were displayed the same, the actual values were different, which led to different rankings. The average ranking of IMGO’s performance on *f*14−*f*23 is 1.4, which is the best among all algorithms.

**Fig 5:**
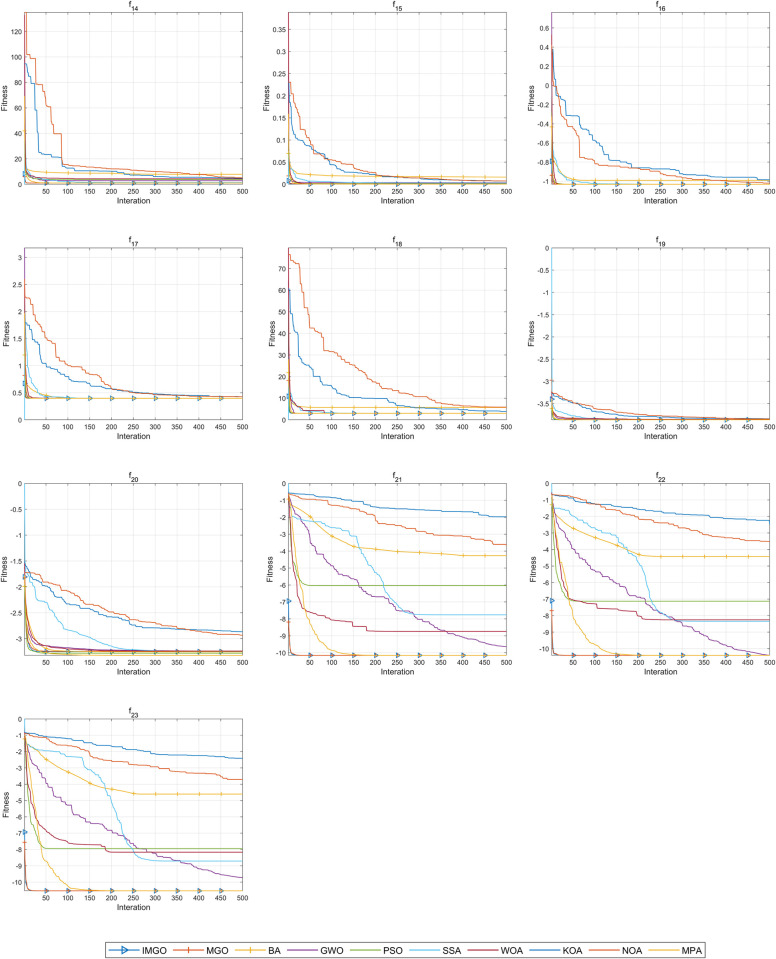
Convergence curves of the IMGO algorithm and comparison algorithms on functions *f*14−*f*23.

The convergence curves for functions *f*14−*f*23 are shown in Fig 5. The IMGO algorithm generally exhibited faster convergence and better optimal solution findings across most benchmark functions. This algorithm converged more quickly to the optimal solutions on functions *f*14, *f*16, *f*17, *f*18, *f*19, *f*21, *f*22, *f*23 than on the other functions. Overall, the IMGO demonstrated the best performance on functions *f*14−*f*23.

The experimental results on the multimodal benchmark functions (*f*8−*f*23) prove that the IMGO achieved a balance between exploration and development and avoided becoming trapped in local optimal solutions. The IMGO method yielded better results in handling both unimodal and multimodal problems because there are many improvements with respect to its ability to balance exploration and exploitation.

The 5% Wilcoxon rank-sum test statistics for IMGO and the other compared algorithms on each benchmark function are presented in Table 7. Table 7 presents these results, employing the symbols (+, =, and −) to denote whether IMGO’s performance on each benchmark function is ’significantly better than, comparable to, or worse than’ that of the other algorithms under comparison.

**Table 7:** The Wilcoxon rank-sum statistical test results on the 23 basic benchmark functions.

Function	BA		GWO		PSO		SSA		WOA		KOA		NOA		MPA		MGO	
***f*1**	3.40E-08	+	3.40E-08	+	3.40E-08	+	3.40E-08	+	3.95E-08	+	3.40E-08	+	3.40E-08	+	3.40E-08	+	3.40E-08	+
***f*2**	3.40E-08	+	3.40E-08	+	3.40E-08	+	3.40E-08	+	1.00E+00	-	3.40E-08	+	3.40E-08	+	3.40E-08	+	9.59E-08	+
***f*3**	3.40E-08	+	3.40E-08	+	3.40E-08	+	3.40E-08	+	3.40E-08	+	3.40E-08	+	1.00E+00	-	3.40E-08	+	3.02E-03	+
***f*4**	3.40E-08	+	3.40E-08	+	3.40E-08	+	3.40E-08	+	3.40E-08	+	3.40E-08	+	3.40E-08	+	3.40E-08	+	3.40E-08	+
***f*5**	5.63E-09	+	5.63E-09	+	5.63E-09	+	5.63E-09	+	5.63E-09	+	5.63E-09	+	5.63E-09	+	5.63E-09	+	3.55E-06	+
***f*6**	3.40E-08	+	3.40E-08	=	9.97E-01	+	2.08E-01	-	3.40E-08	+	3.40E-08	+	3.40E-08	+	9.95E-01	-	1.00E+00	-
***f*7**	3.40E-08	+	3.95E-08	+	3.40E-08	+	3.40E-08	+	1.71E-07	+	3.40E-08	+	3.95E-08	+	3.40E-08	+	1.52E-04	+
***f*8**	1.00E+00	-	3.40E-08	+	3.40E-08	+	3.40E-08	+	3.40E-08	+	3.40E-08	+	3.40E-08	+	3.40E-08	+	1.00E+00	-
***f*9**	4.00E-09	+	3.98E-09	+	4.00E-09	+	4.00E-09	+	3.42E-01	=	4.00E-09	+	8.06E-02	=	NaN	=	NaN	=
***f*10**	7.56E-09	+	7.18E-09	+	7.56E-09	+	7.56E-09	+	5.67E-06	+	7.55E-09	+	8.17E-03	+	7.56E-09	+	3.94E-01	=
***f*11**	4.00E-09	+	1.63E-01	=	4.00E-09	+	4.00E-09	+	3.42E-01	=	4.00E-09	+	2.27E-03	+	NaN	=	NaN	=
***f*12**	3.40E-08	+	3.40E-08	+	3.40E-08	+	3.40E-08	+	3.40E-08	+	3.40E-08	+	3.40E-08	+	3.40E-08	+	1.00E+00	-
***f*13**	4.00E-09	+	4.00E-09	+	4.00E-09	+	4.00E-09	+	4.00E-09	+	4.00E-09	+	4.00E-09	+	4.00E-09	+	1.02E-03	+
***f*14**	1.93E-08	+	1.94E-08	+	7.85E-07	+	6.54E-05	+	1.94E-08	+	1.94E-08	+	1.94E-08	+	1.00E-02	+	6.71E-04	+
***f*15**	3.40E-08	+	3.40E-08	+	5.65E-02	=	3.40E-08	+	3.40E-08	+	3.40E-08	+	3.40E-08	+	1.00E+00	-	8.59E-02	=
***f*16**	1.43E-08	+	1.43E-08	+	4.47E-01	=	1.43E-08	+	1.43E-08	+	1.43E-08	+	1.43E-08	+	7.13E-07	+	3.56E-06	+
***f*17**	5.63E-09	+	5.63E-09	+	3.42E-01	=	4.34E-06	+	5.63E-09	+	5.63E-09	+	5.63E-09	+	3.42E-01	=	3.42E-01	=
***f*18**	3.35E-08	+	3.35E-08	+	1.00E+00	+	3.35E-08	-	3.35E-08	+	3.35E-08	+	3.35E-08	+	1.00E+00	-	1.00E+00	-
***f*19**	2.07E-08	+	2.07E-08	+	1.00E+00	-	2.07E-08	+	2.07E-08	+	2.07E-08	+	2.07E-08	+	3.56E-01	=	1.06E-03	+
***f*20**	6.50E-03	+	6.50E-03	+	1.00E+00	+	2.48E-03	=	1.32E-01	=	3.05E-08	+	1.79E-07	+	2.84E-01	=	7.21E-02	=
***f*21**	2.52E-08	+	2.52E-08	+	2.43E-01	=	2.52E-08	+	2.52E-08	+	2.52E-08	+	2.52E-08	+	2.52E-08	+	1.00E+00	-
***f*22**	2.70E-08	+	2.70E-08	+	8.34E-01	=	2.70E-08	+	2.70E-08	+	2.70E-08	+	2.70E-08	+	2.70E-08	+	1.00E+00	-
***f*23**	2.79E-08	+	2.79E-08	+	5.48E-01	=	2.79E-08	+	2.79E-08	+	2.79E-08	+	2.79E-08	+	2.79E-08	+	1.00E+00	-
**+/ = /-**	22/0/1		21/2/0		16/6/1		20/1/2		19/3/0		23/0/0		21/1/1		15/5/3		10/6/7	

Table 7 indicates that the IMGO algorithm outperformed the other algorithms in most cases: it outperformed the BA on 22 out of 23 functions, the GWO algorithm on 21 out of 23 functions, the PSO algorithm on 16 functions, the SSA on 20 functions, the WOA on 19 functions, the KOA algorithm on all 23 functions, the NOA algorithm on 21 functions, the MPA on 15 functions, and the MGO algorithm on 10 functions. However, this algorithm was less effective than the BA on 1 function, the PSO on 1 function, the SSA on 2 functions, NOA on 1 function, and the MPA on 3 functions. Compared to the performance of the MGO, the IMGO underperformed on 7 of 23 functions. The statistical results indicated that the median performance of IMGO was the best among all algorithms.

In conclusion, the comparative analysis of the IMGO against competing algorithms across 23 benchmark functions revealed that the IMGO not only outperformed the original MGO algorithm but also outperformed the other comparative methods. The average ranking of IMGO is 1.4, which is the best ranking among all algorithms.

The IMGO algorithm outperformed the original MGO algorithm on 16 of 23 functions, while it inferior to the MGO algorithm on only 2 functions. This finding underscores the fact that our improvements are both valid and efficacious, substantially boosting its optimization and convergence capacities. The experiment shows that IMGO has excellent optimization ability in dealing with continuous space problems.

### B. Feature selection from the medical dataset

The BIMGO algorithm is compared with 8 binary optimization algorithms on medical datasets, namely, the BGWO, BSSA, BBA, BPSO, BMPA, BNOA, BKOA, and BWOA algorithms.

The parameters are set based on their original papers, as shown in Table 2. To ensure fairness in the evaluation, all algorithms use the same common parameters on medical datasets. These parameters include a population size of 30 individuals, a maximum iterative number of 100 times, and 20 independent runs for each algorithm. A support vector machine (SVM) classifier [61] is utilized to classify the feature subsets generated by all algorithms to evaluate the performance of all algorithms.

#### 1) Dataset

The datasets used here are 16 standard medical datasets from the UCI Machine Learning Repository and OPENML [62–73]. The structure, including the numbers of instances and features and the presence of missing values, is detailed in Table 8.

**Table 8:** Details of the medical datasets.

No.	Datasets	Instances	Features	Missing values
**1**	Heart Disease	303	13	Yes
**2**	Breast Cancer	569	30	No
**3**	ILPD	583	10	No
**4**	SPECT	267	22	No
**5**	Cervical Cancer	858	35	Yes
**6**	Parkinsons Ⅰ	197	23	No
**7**	Thoracic Surgery Data	470	16	No
**8**	Glioma	839	23	No
**9**	Bone Marrow Transplant	187	36	Yes
**10**	Diabetes	520	16	Yes
**11**	Parkinson Ⅱ	756	754	No
**12**	Colon	62	2000	No
**13**	Leukemia	72	7129	No
**14**	Arcene Cancer Dataset	200	10000	No
**15**	DLBCL	77	5469	No
**16**	Prostate Tumors	102	12600	No

#### 2) Evaluation metrics

The evaluation criteria are set as the fitness, sensitivity, and the number of selected features.

Fitness is measured by the error rate in classifying medical datasets, calculated using Eq (17). The sensitivity represents how many true positives are correctly classified. For medical datasets, the sensitivity can reflect the algorithm’s ability to correctly detect disease features. The sensitivity is an important indicator and is calculated using Eq (18).

$$Fitness=1-Accuracy=1-\frac{TP+TN}{TP+TN+FP+FN}$$ (17)

$$Sensitivity=\frac{TP}{TP+FN}$$ (18)

where, *TP*, *TN*, *FP*, and *FN* denote true positives, true negatives, false positives and false negatives, respectively.

#### 3) Experimental results

The performance of the BIMGO was assessed on the target medical datasets and compared with that of the eight well-known methods. Tables 9–11 show the results of the experiment and the ranks of all the algorithms based on the mean values. The best values are highlighted in bold.

**Table 9:** Comparison in terms of the fitness values of the algorithms.

Datasets		BBA	BGWO	BPSO	BSSA	BWOA	BKOA	BNOA	BMPA	BIMGO
***Bone***	Mean	0.2083	0.0397	0.1909	0.0854	0.0964	0.0465	0.0743	0.0632	0.0255
	Std	0.0750	0.0130	0.0700	0.0356	0.0131	0.0175	0.0210	0.0191	0.0166
	Min	0.0574	0.0272	0.0557	0.0302	0.0812	0.0283	0.0540	0.0288	0.0006
	Rank	9	2	8	6	7	3	5	4	**1**
***Breast Cancer***	Mean	0.0455	0.0280	0.0362	0.0176	0.0280	0.0250	0.0592	0.0043	0.0128
	Std	0.0070	0.0034	0.0074	0.0039	0.0001	0.0038	0.0051	0.0016	0.0007
	Min	0.0324	0.0197	0.0217	0.0127	0.0277	0.0210	0.0491	0.0030	0.0114
	Rank	8	5	7	3	6	4	9	**1**	2
***Cervical Cancer***	Mean	0.0341	0.0284	0.0739	0.0371	0.0348	0.0264	0.0384	0.0198	0.0143
	Std	0.0009	0.0123	0.0096	0.0019	0.0102	0.0015	0.0004	0.0043	0.0004
	Min	0.0328	0.0181	0.0509	0.0345	0.0176	0.0247	0.0374	0.0144	0.0135
	Rank	5	4	9	7	6	3	8	2	**1**
***Diabetes***	Mean	0.0824	0.0311	0.0615	0.0577	0.0337	0.0322	0.0591	0.0270	0.0514
	Std	0.0083	0.0067	0.0076	0.0042	0.0007	0.0045	0.0084	0.0046	0.0079
	Min	0.0716	0.0228	0.0545	0.0551	0.0329	0.0253	0.0443	0.0247	0.0450
	Rank	9	2	8	6	4	3	7	**1**	5
***Glioma***	Mean	0.1404	0.1428	0.1381	0.1692	0.1502	0.1272	0.1566	0.1274	0.1187
	Std	0.0017	0.0001	0.0126	0.0005	0.0003	0.0007	0.0005	0.0008	0.0000
	Min	0.1362	0.1427	0.1309	0.1685	0.1495	0.1255	0.1554	0.1259	0.1187
	Rank	5	6	4	9	7	2	8	3	**1**
***Heart Disease***	Mean	0.0759	0.1401	0.1713	0.1498	0.1256	0.2115	0.1404	0.1154	0.0893
	Std	0.0094	0.0100	0.0106	0.0080	0.0079	0.0107	0.0103	0.0193	0.0005
	Min	0.0564	0.1217	0.1531	0.1374	0.1186	0.1869	0.1217	0.0698	0.0871
	Rank	**1**	5	8	7	4	9	6	3	2
***Parkinsons Ⅰ***	Mean	0.1388	0.0518	0.0508	0.1820	0.0781	0.1342	0.0986	0.0522	0.0518
	Std	0.0160	0.0002	0.0095	0.0006	0.0003	0.0092	0.0108	0.0002	0.0003
	Min	0.1070	0.0517	0.0286	0.1809	0.0775	0.1292	0.0784	0.0521	0.0517
	Rank	8	2	**1**	9	5	7	6	4	3
***ILPD***	Mean	0.2776	0.2912	0.2829	0.2575	0.3338	0.3056	0.2745	0.2503	0.2485
	Std	0.0014	0.0000	0.0233	0.0005	0.0000	0.0102	0.0005	0.0011	0.0000
	Min	0.2751	0.2912	0.2485	0.2570	0.3338	0.2630	0.2741	0.2485	0.2485
	Rank	5	7	6	3	9	8	4	2	**1**
***SPECT***	Mean	0.1504	0.1430	0.0649	0.1425	0.0959	0.1563	0.1403	0.1007	0.0626
	Std	0.0211	0.0084	0.0081	0.0092	0.0007	0.0128	0.0108	0.0066	0.0068
	Min	0.1141	0.1324	0.0591	0.1329	0.0948	0.1329	0.1150	0.0958	0.0595
	Rank	8	7	2	6	3	9	5	4	**1**
***Thoracic Surgery***	Mean	0.1839	0.1270	0.1190	0.1077	0.1694	0.1804	0.1489	0.1481	0.1165
	Std	0.0013	0.0000	0.0012	0.0005	0.0003	0.0006	0.0007	0.0000	0.0000
	Min	0.1803	0.1270	0.1165	0.1066	0.1691	0.1797	0.1481	0.1481	0.1165
	Rank	9	4	3	**1**	7	8	6	5	2
***Arcene Cancer***	Mean	0.0119	0.0062	0.0131	0.0253	0.0084	0.0163	0.0178	0.0050	0.0003
	Std	0.0044	0.0000	0.0027	0.0025	0.0031	0.0031	0.0023	0.0026	0.0014
	Min	0.0062	0.0062	0.0125	0.0188	0.0062	0.0125	0.0125	0.0000	0.0000
	Rank	5	3	6	9	4	7	8	2	**1**
***Colon***	Mean	0.0200	0.0400	0.0200	0.0000	0.0200	0.0600	0.0400	0.0280	0.0053
	Std	0.0000	0.0000	0.0000	0.0000	0.0000	0.0000	0.0000	0.0101	0.0090
	Min	0.0200	0.0400	0.0200	0.0000	0.0200	0.0600	0.0400	0.0200	0.0000
	Rank	3	7	3	**1**	3	9	7	6	2
***DLBCL***	Mean	0.0089	0.0056	0.0000	0.0040	0.0000	0.0000	0.0000	0.0000	0.0000
	Std	0.0152	0.0079	0.0000	0.0072	0.0000	0.0000	0.0000	0.0000	0.0000
	Min	0.0000	0.0000	0.0000	0.0000	0.0000	0.0000	0.0000	0.0000	0.0000
	Rank	9	8	**1**	7	**1**	**1**	**1**	**1**	**1**
***Parkinson Ⅱ***	Mean	0.2529	0.2727	0.2645	0.2669	0.2480	0.2397	0.2520	0.2518	0.1938
	Std	0.0000	0.0000	0.0000	0.0076	0.0025	0.0000	0.0076	0.0057	0.0228
	Min	0.2529	0.2727	0.2645	0.2446	0.2446	0.2397	0.2347	0.2380	0.1570
	Rank	6	9	7	8	3	2	5	4	**1**
***Prostate Tumors***	Mean	0.0323	0.0018	0.0000	0.0000	0.0061	0.0000	0.0000	0.0000	0.0000
	Std	0.0091	0.0045	0.0000	0.0000	0.0074	0.0000	0.0000	0.0000	0.0000
	Min	0.0122	0.0000	0.0000	0.0000	0.0000	0.0000	0.0000	0.0000	0.0000
	Rank	9	7	**1**	**1**	8	**1**	**1**	**1**	**1**
***Leukemia***	Mean	0.0043	0.0000	0.0000	0.0000	0.0000	0.0000	0.0007	0.0000	0.0000
	Std	0.0077	0.0000	0.0000	0.0000	0.0000	0.0000	0.0036	0.0000	0.0000
	Min	0.0000	0.0000	0.0000	0.0000	0.0000	0.0000	0.0000	0.0000	0.0000
	Rank	9	**1**	**1**	**1**	**1**	**1**	8	**1**	**1**
**Average rank**		6.8	4.9	4.7	5.3	4.9	4.8	5.9	2.8	**1.6**

**Table 10:** Comparison in terms of the sensitivity.

Datasets		BBA	BGWO	BPSO	BSSA	BWOA	BKOA	BNOA	BMPA	BIMGO
***Bone***	Mean	0.3471	0.8029	0.6618	0.6882	0.8059	0.7706	0.7647	0.7618	0.8294
	Std	0.2588	0.0478	0.1361	0.1719	0.0471	0.0830	0.0540	0.0864	0.0536
	Max	0.7647	0.8824	0.8235	0.8824	0.8824	0.9412	0.8824	0.8824	0.9412
	Rank	9	3	8	7	2	4	5	6	**1**
***Breast Cancer***	Mean	0.9250	0.9316	0.9276	0.9421	0.9026	0.9079	0.9579	0.8974	0.9171
	Std	0.0323	0.0493	0.0306	0.0202	0.0124	0.0160	0.0179	0.0081	0.0196
	Max	1.0000	1.0000	1.0000	0.9737	0.9211	0.9474	0.9737	0.9211	0.9474
	Rank	5	3	4	2	8	7	**1**	9	6
***Cervical Cancer***	Mean	0.2889	0.6222	0.3556	0.7444	0.7778	0.7778	0.1111	0.8889	0.8889
	Std	0.2981	0.4179	0.3236	0.1026	0.1974	0.0000	0.0883	0.0000	0.0000
	Max	0.7778	0.8889	0.8889	0.8889	0.8889	0.7778	0.3333	0.8889	0.8889
	Rank	8	6	7	5	3	3	9	**1**	**1**
***Diabetes***	Mean	0.8966	0.9341	0.9284	0.9477	0.9807	0.9330	0.9216	0.9170	0.9489
	Std	0.0407	0.0102	0.0152	0.0377	0.0288	0.0051	0.0393	0.0212	0.0303
	Max	0.9545	0.9545	0.9318	0.9773	1.0000	0.9545	1.0000	1.0000	1.0000
	Rank	9	4	6	3	**1**	5	7	8	2
***Glioma***	Mean	0.8953	0.9063	0.8984	0.9063	0.9063	0.9063	0.9063	0.9063	0.9063
	Std	0.0269	0.0000	0.0261	0.0000	0.0000	0.0000	0.0000	0.0000	0.0000
	Max	0.9063	0.9063	0.9063	0.9063	0.9063	0.9063	0.9063	0.9063	0.9063
	Rank	9	**1**	8	**1**	**1**	**1**	**1**	**1**	**1**
***Heart Disease***	Mean	0.8917	0.8750	0.8479	0.8750	0.8354	0.8750	0.8833	0.8542	0.8792
	Std	0.0209	0.0427	0.0511	0.0000	0.0093	0.0427	0.0256	0.0345	0.0355
	Max	0.9167	0.9167	0.9167	0.8750	0.8750	0.9583	0.9167	0.8750	0.9167
	Rank	**1**	4	8	4	9	4	2	7	3
***Parkinsons Ⅰ***	Mean	0.9194	0.9532	0.9823	0.9581	0.9113	0.8952	0.9016	0.9597	0.9661
	Std	0.0339	0.0355	0.0286	0.0379	0.0430	0.0274	0.0412	0.0455	0.0072
	Max	1.0000	1.0000	1.0000	1.0000	1.0000	0.9677	1.0000	1.0000	0.9677
	Rank	6	5	**1**	4	7	9	8	3	2
***ILPD***	Mean	1.0000	1.0000	1.0000	1.0000	1.0000	1.0000	1.0000	1.0000	1.0000
	Std	0.0000	0.0000	0.0000	0.0000	0.0000	0.0000	0.0000	0.0000	0.0000
	Max	1.0000	1.0000	1.0000	1.0000	1.0000	1.0000	1.0000	1.0000	1.0000
	Rank	**1**	**1**	**1**	**1**	**1**	**1**	**1**	**1**	**1**
***SPECT***	Mean	0.8989	0.9216	0.9295	0.9148	0.9341	0.9205	0.9045	0.8784	0.9432
	Std	0.0497	0.0281	0.0102	0.0243	0.0243	0.0271	0.0351	0.0474	0.0117
	Max	1.0000	1.0000	0.9545	0.9545	0.9773	0.9773	1.0000	0.9545	0.9545
	Rank	8	4	3	6	2	5	7	9	**1**
***Thoracic Surgery***	Mean	1.0000	1.0000	1.0000	1.0000	1.0000	1.0000	1.0000	1.0000	1.0000
	Std	0.0000	0.0000	0.0000	0.0000	0.0000	0.0000	0.0000	0.0000	0.0000
	Max	1.0000	1.0000	1.0000	1.0000	1.0000	1.0000	1.0000	1.0000	1.0000
	Rank	**1**	**1**	**1**	**1**	**1**	**1**	**1**	**1**	**1**
***Arcene Cancer***	Mean	0.8042	0.7910	0.7751	0.8042	0.7884	0.7937	0.7672	0.7910	0.8333
	Std	0.0516	0.0841	0.0814	0.0672	0.0817	0.0612	0.0624	0.0655	0.0430
	Max	0.8889	0.8889	0.8889	0.8889	0.9444	0.8889	0.8333	0.8889	0.8889
	Rank	2	5	8	2	7	4	9	5	**1**
***Colon***	Mean	0.7500	0.7500	0.7500	0.7500	0.7500	0.7500	0.7500	0.7500	0.7875
	Std	0.0000	0.0000	0.0000	0.0000	0.0000	0.0000	0.0000	0.0000	0.0916
	Max	0.7500	0.7500	0.7500	0.7500	0.7500	0.7500	0.7500	0.7500	1.0000
	Rank	2	2	2	2	2	2	2	2	**1**
***DLBCL***	Mean	0.6700	0.8400	0.8700	0.9200	0.8800	0.8300	0.8200	0.8500	0.9200
	Std	0.3570	0.1903	0.2080	0.1361	0.1508	0.2273	0.2042	0.2417	0.1989
	Max	1.0000	1.0000	1.0000	1.0000	1.0000	1.0000	1.0000	1.0000	1.0000
	Rank	9	6	4	**1**	3	7	8	5	**1**
***Parkinson Ⅱ***	mean	0.7814	1.0000	1.0000	1.0000	0.9000	0.6137	1.0000	0.7969	0.9407
	std	0.2888	0.0000	0.0000	0.0000	0.3078	0.4621	0.0000	0.4090	0.2226
	max	1.0000	1.0000	1.0000	1.0000	1.0000	0.9469	1.0000	1.0000	1.0000
	Rank	8	**1**	**1**	1	6	9	**1**	7	5
***Prostate Tumors***	Mean	1.0000	1.0000	1.0000	1.0000	1.0000	0.9958	1.0000	1.0000	1.0000
	Std	0.0000	0.0000	0.0000	0.0000	0.0000	0.0186	0.0000	0.0000	0.0000
	Max	1.0000	1.0000	1.0000	1.0000	1.0000	1.0000	1.0000	1.0000	1.0000
	Rank	**1**	**1**	**1**	**1**	**1**	9	**1**	**1**	**1**
***Leukemia***	Mean	0.9318	0.8682	0.9182	0.9909	0.9455	1.0000	0.9129	0.9394	0.8818
	Std	0.1247	0.2528	0.1530	0.0280	0.1515	0.0000	0.2136	0.1276	0.2434
	Max	1.0000	1.0000	1.0000	1.0000	1.0000	1.0000	1.0000	1.0000	1.0000
	Rank	5	9	6	2	3	**1**	7	4	8
**Average rank**		5.3	3.5	4.3	2.7	3.6	4.5	4.4	4.4	**2.3**

**Table 11:** Comparison in terms of the selected features.

Datasets		BBA	BGWO	BPSO	BSSA	BWOA	BKOA	BNOA	BMPA	BIMGO
***Bone***	Mean	18.25	7.10	16.80	21.40	14.20	12.70	14.15	11.75	6.85
	Std	2.49	3.58	3.49	3.84	2.95	2.85	4.66	3.80	5.23
	Min	12	2	11	15	9	8	7	4	2
	Rank	8	2	7	9	6	4	5	3	**1**
***Breast Cancer***	Mean	15.40	7.25	14.95	15.15	5.95	13.75	13.35	11.55	12.40
	Std	2.76	1.80	3.00	2.78	0.39	2.67	2.68	1.85	2.04
	Min	11	5	12	12	5	10	8	6	8
	Rank	9	2	7	8	**1**	6	5	3	4
***Cervical Cancer***	Mean	18.75	2.65	14.80	18.90	1.00	10.75	13.60	5.85	9.95
	Std	3.09	1.50	2.71	4.53	0.00	2.75	1.54	5.59	1.54
	Min	14	1	9	12	1	6	10	1	7
	Rank	8	2	7	9	**1**	5	6	3	4
***Diabetes***	Mean	11.45	10.15	11.60	12.40	8.30	8.95	10.10	9.65	11.80
	Std	2.21	2.41	1.57	0.75	1.13	2.09	1.77	1.60	1.44
	Min	6	6	8	11	7	5	7	8	10
	Rank	6	5	7	9	**1**	2	4	3	8
***Glioma***	Mean	12.55	3.10	3.75	9.65	6.70	7.85	7.70	8.50	2.00
	Std	2.63	0.31	2.45	1.09	0.80	1.63	1.22	1.79	0.00
	Min	8	3	3	8	5	4	5	5	2
	Rank	9	2	3	8	4	6	5	7	**1**
***Heart Disease***	Mean	8.60	6.25	8.15	8.15	4.50	5.75	7.65	5.90	8.85
	Std	1.43	1.65	1.42	0.88	1.36	1.74	1.69	1.52	0.67
	Min	6	4	6	7	3	1	5	4	6
	Rank	8	4	6	6	**1**	2	5	3	9
***Parkinsons Ⅰ***	Mean	12.15	2.20	8.50	9.40	4.25	7.70	7.40	3.25	2.30
	Std	2.30	0.52	1.43	1.35	0.55	2.00	2.09	0.44	0.66
	Min	9	2	6	7	3	5	4	3	2
	Rank	9	**1**	7	8	4	6	5	3	2
***ILPD***	Mean	4.45	1.00	2.35	1.50	1.00	1.75	1.35	2.75	1.00
	Std	1.39	0.00	3.30	0.51	0.00	2.17	0.49	1.07	0.00
	Min	2	1	1	1	1	1	1	1	1
	Rank	9	**1**	7	5	**1**	6	4	8	**1**
***SPECT***	Mean	12.15	8.00	11.60	13.00	9.25	11.10	10.25	11.80	10.35
	Std	2.50	2.87	1.79	1.65	1.45	1.74	2.02	2.98	0.59
	Min	7	4	9	10	7	9	6	7	9
	Rank	8	**1**	6	9	2	5	3	7	4
***Thoracic Surgery***	Mean	7.70	1.00	5.05	3.80	1.35	2.10	2.35	1.00	1.00
	Std	2.03	0.00	1.90	0.77	0.49	0.91	1.14	0.00	0.00
	Min	2	1	1	2	1	1	1	1	1
	Rank	9	**1**	8	7	4	5	6	**1**	**1**
***Arcene Cancer***	Mean	5004.00	8664.20	9393.86	7623.75	5920.45	8855.65	4936.25	8412.75	3853.95
	Std	44.39	390.22	366.97	1657.02	1398.25	1973.89	190.04	1150.27	1579.63
	Min	4917	8394	8426	6172	4902	3163	4187	5401	796
	Rank	3	7	9	5	4	8	2	6	**1**
***Colon***	Mean	1002.65	2000.00	1891.45	1265.50	1510.65	2000.00	993.20	1648.35	333.55
	Std	21.48	0.00	23.07	46.79	21.46	0.00	18.02	329.02	391.68
	Min	974	2000	1864	1215	1472	2000	966	882	41
	Rank	3	8	7	4	5	8	2	6	**1**
***DLBCL***	Mean	2885.20	4682.05	5142.70	4024.00	2847.60	5469.00	2924.55	4616.14	3290.90
	Std	342.97	100.97	286.28	514.43	458.45	0.00	529.07	258.28	1117.89
	Min	2690	4564	4653	3407	2642	5469	2278	4076	2673
	Rank	2	7	8	5	**1**	9	3	6	4
***Parkinson Ⅱ***	Mean	375.65	754.00	754.00	727.25	381.45	754.00	378.10	704.25	14.95
	Std	16.27	0.00	0.00	82.42	12.93	0.00	23.18	106.29	10.38
	Min	338	754	754	475	346	754	296	415	3
	Rank	2	7	7	6	4	7	3	5	**1**
***Prostate Tumors***	Mean	6306.60	10725.40	12288.85	7902.05	4114.30	11947.40	6478.75	10784.30	6881.50
	Std	49.98	147.31	505.13	52.20	2311.01	1018.20	714.43	582.94	679.34
	Min	6209	10424	10771	7819	953	7894	6223	9549	6297
	Rank	2	6	9	5	**1**	8	3	7	4
***Leukemia***	Mean	3646.95	6302.00	6886.65	4824.60	3845.80	7129.00	3790.35	6393.65	3785.65
	Std	230.68	307.34	267.54	909.19	517.85	0.00	636.44	50.93	524.74
	Min	3499	5998	5920	4392	3546	7129	2854	6335	3098
	Rank	**1**	6	8	5	4	9	3	7	2
**Average rank**		6.0	3.9	7.1	6.8	**2.8**	6.0	4.0	4.9	3.0

A comparison of the performance of the BIMGO algorithm and that of the comparison algorithms in terms of fitness values is shown in Table 9. The BIMGO obtained the best average fitness values on 10 out of 16 datasets, namely Bone Marrow Transplant, Cervical Cancer, Glioma, ILPD, SPECT, Arcene Cancer, DLBCL, Parkinson Ⅱ, Prostate Tumors, and Leukemia. That is, BIMGO achieved the best average classification accuracy on the 10 datasets mentioned above. The proposed algorithm slightly underperformed compared to BMPA on the Breast Cancer dataset. The BIMGO achieved moderate performance on the Diabetes dataset, ranked only the fifth. While the BIMGO slightly underperformed compared to the BBA on the Heart Disease dataset, it achieved significantly better performance than the comparison algorithms. On the Parkinson Ⅰ dataset, the BIMGO algorithm did not perform as well as the BPSO and BGWO. On the Thoracic Surgery and the Colon datasets, the BIMGO ranked second to the BSSA. Overall, the BIMGO achieved an impressive average rank of 1.6 across 16 medical datasets, which is significantly better than other optimization algorithms.

**Fig 6:**
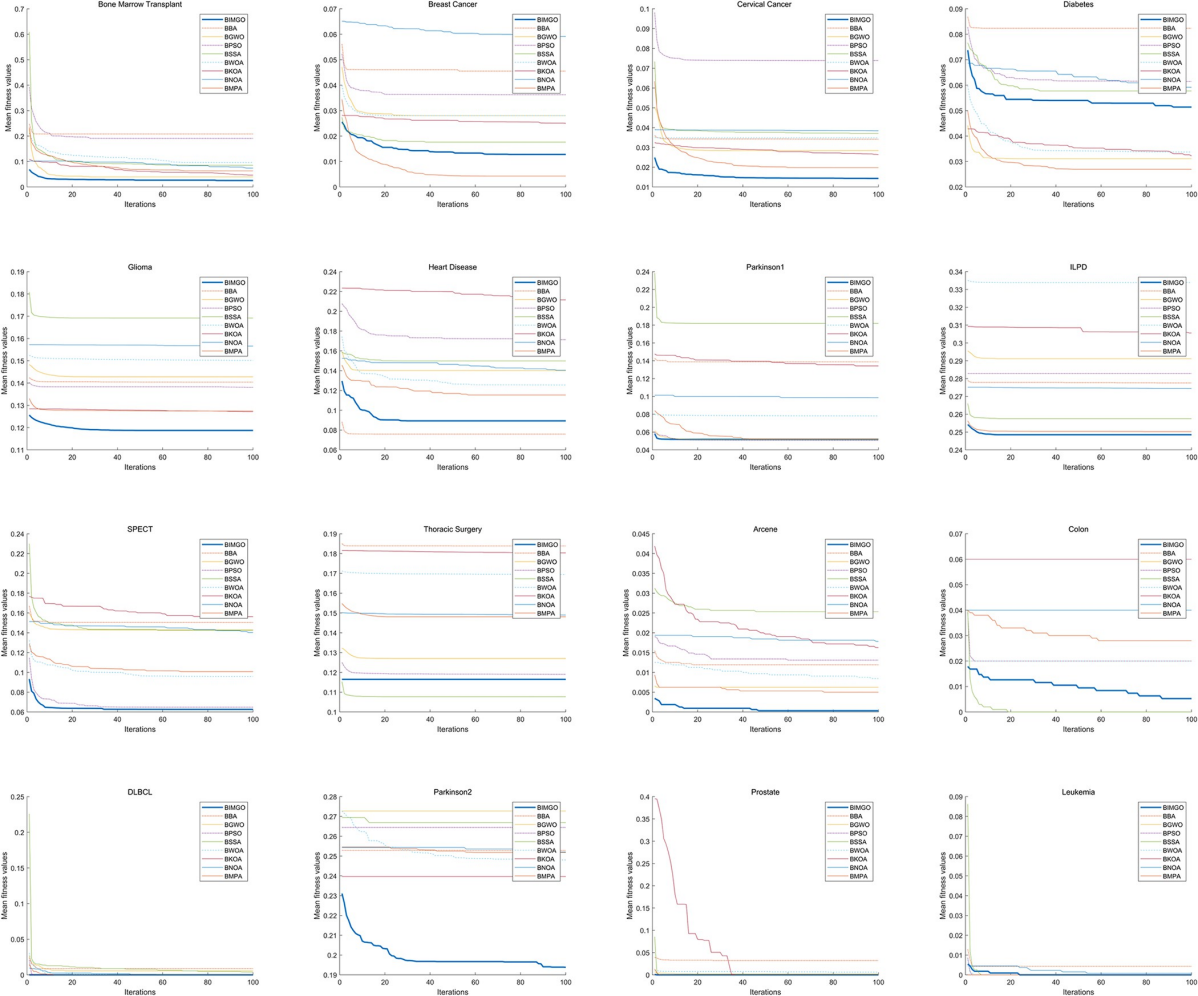
Comparative analysis of algorithm convergence on medical datasets.

Fig 6 plots the convergence curve for each algorithm based on the fitness value. The proposed BIMGO exhibited significantly better convergence and fitness values than other algorithms on the Bone, Cervical Cancer, Glioma, Arcene Cancer, and Parkinson II datasets, and exhibited strong convergence with slightly better fitness values than other algorithms on the ILPD and SPECT datasets. Multiple algorithms achieved the best fitness values on DLBCL and Prostate datasets, but BIMGO had the strongest convergence ability. Although BIMGO also achieved the best fitness value on the Leukemia dataset, its convergence ability is average. BIMGO demonstrated excellent convergence ability and achieved the 2nd best fitness value on the Breast Cancer, Heart Disease, Prostate, and Colon datasets. Although BIMGO only achieved the third fitness value on the Parkinsons I dataset, the gap with BPSO and BGWO algorithms is very small and the convergence ability is excellent. The proposed algorithm exhibited average convergence ability and fitness value on the Diabetes dataset. It is shown in Fig 6 that comparing with the competing algorithms, the BIMGO algorithm gains the best overall performance on 10 data sets.

Table 10 presents a comparison of the sensitivity, expressed in percentage. The BIMGO achieved the highest sensitivity on 10 of the 16 datasets. BIMGO’s performance on the Breast Cancer dataset was average, ranking 6th. On the Diabetes dataset, the BIMGO slightly underperformed compared to BWOA. BIMGO was slightly worse than BBA and BNOA on the Heart Disease dataset, and BPSO on the Parkinsons I dataset. All algorithms accurately found all positive samples on the Thoracic Surgery dataset. On the Parkinson II dataset, BIMGO ranked 5th and obtained the average sensitivity of 94.07%. BIMGO ranked 8th on the Leukemia dataset, with a large gap between the sensitivity achieved by algorithms such as BKOA. The BIMGO maintained the highest average sensitivity ranking across all datasets, highlighting its superior ability to accurately detect disease features.

**Fig 7:**
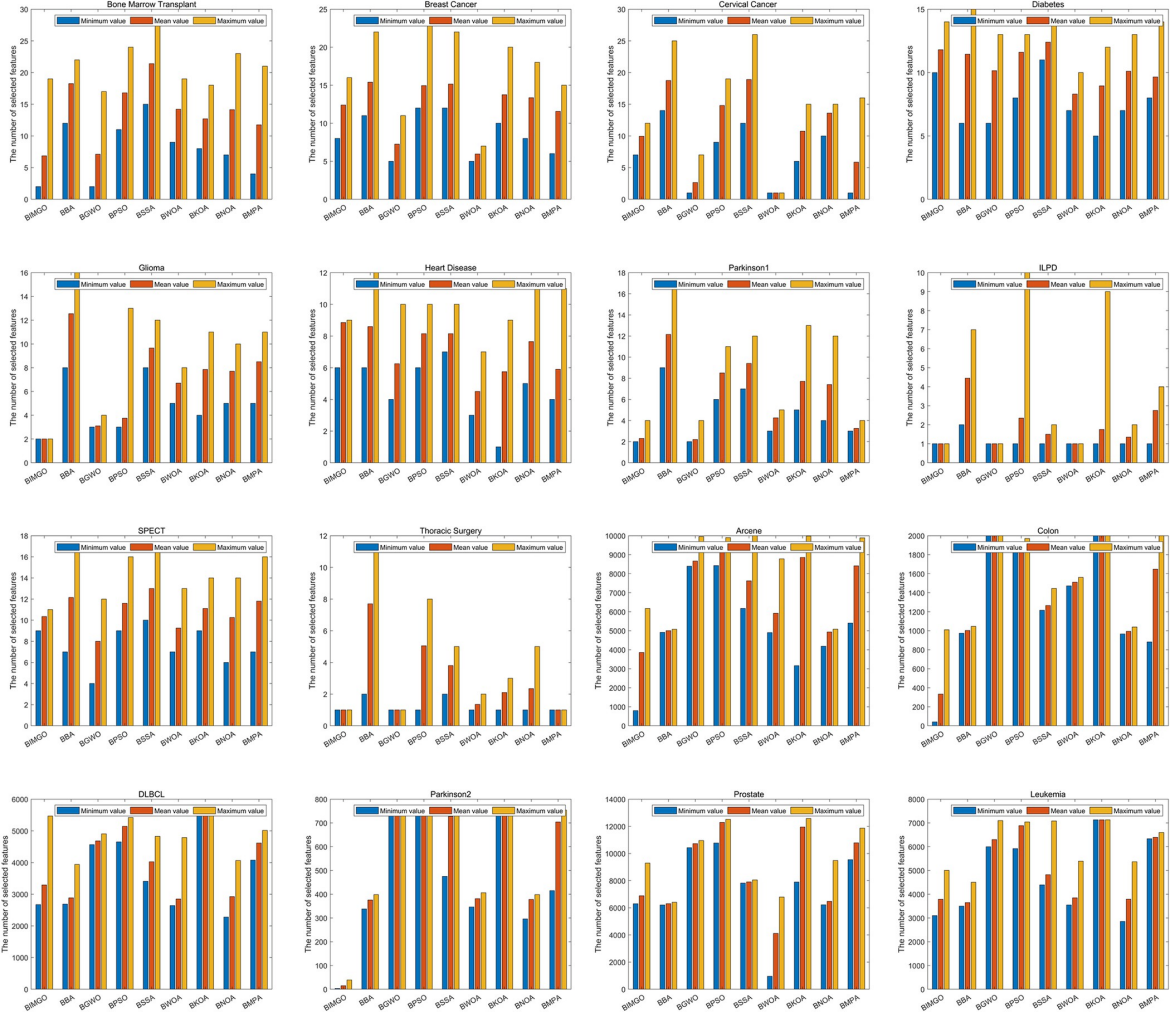
Comparative analysis of feature numbers on medical datasets.

We also compared the number of features selected by different algorithms. Table 11 the details of the results. An intuitive comparison is shown in Fig 7.

On average, in terms of the number of features selected, the BIMGO ranked second across the sixteen medical datasets, as shown in Table 11. This algorithm selected fewer features while maintaining the best classification accuracy, demonstrating its effectiveness in FS for medical datasets.

Fig 7 shows the maximum, minimum, and average number of features selected by these 9 algorithms for feature selection on 16 medical datasets. In this paper, the average number of selected features was mainly used as the evaluation criterion. From Fig 7, BIMGO achieved the lowest average number of features on the Bone, Glioma, ILPD, Thoracic Surge, Arcene Cancer, Colon, and Parkinson II datasets. In particular, the feature approximation rate of BIMGO was much larger than other algorithms on Glioma, Colon and Parkinson Ⅱ datasets. On the Parkinsons Ⅰ, SPECT, DLBCL, and Leukemia datasets, the average number of features selected by BIMGO was not the least, but it is not far from the algorithms selecting fewer features. There was a certain gap of the average number of features selected between BIMGO and better algorithms on the Break Cancer, Diabetes, Heart Disease, and Prostate Tumors datasets. On the Cervical dataset, the average number of features selected by the BIMGO algorithm differed significantly from algorithms such as BWOA.

A 5% Wilcoxon rank-sum test was conducted to statistically determine the presence of significant differences in the algorithm performance on the medical datasets. Table 12 presents these results, employing the symbols (+, =, and −) to denote whether BIMGO’s performance on a dataset is ’significantly better than, comparable to, or worse than’ that of the other algorithms under comparison.

**Table 12:** Comparison in terms of the Wilcoxon rank-sum statistical test with 5% among the BIMGO and the 8 comparison algorithms.

Datasets	BBA		BGWO		BPSO		BSSA		BWOA		BKOA		BNOA		BMPA	
***Bone***	3.24E-08	+	5.29E-05	+	3.24E-08	+	3.30E-07	+	3.22E-08	+	4.71E-06	+	3.24E-08	+	1.05E-07	+
***Breast Cancer***	3.11E-08	+	3.02E-08	+	3.11E-08	+	4.92E-07	+	8.88E-09	+	3.03E-08	+	3.06E-08	+	1.00E+00	-
***Cervical Cancer***	2.91E-08	+	2.71E-08	+	3.01E-08	+	2.97E-08	+	1.25E-08	+	2.88E-08	+	2.53E-08	+	1.73E-05	+
***Diabetes***	2.93E-08	+	1.00E+00	-	5.09E-03	+	8.24E-02	=	1.00E+00	-	1.00E+00	-	1.06E-02	+	1.00E+00	-
***Glioma***	3.91E-09	+	5.51E-10	+	1.33E-09	+	3.08E-09	+	2.28E-09	+	3.52E-09	+	3.48E-09	+	3.64E-09	+
***Heart Disease***	1.00E+00	-	5.19E-09	+	5.34E-09	+	5.06E-09	+	4.65E-09	+	5.34E-09	+	5.44E-09	+	1.77E-06	+
***Parkinsons Ⅰ***	1.20E-08	+	6.68E-01	=	4.69E-05	+	1.10E-08	+	6.25E-09	+	1.15E-08	+	1.13E-08	+	6.07E-06	+
***ILPD***	3.28E-09	+	2.34E-10	+	1.09E-06	+	2.14E-09	+	2.34E-10	+	7.78E-10	+	1.79E-09	+	4.47E-07	+
***SPECT***	2.46E-08	+	2.34E-08	+	5.72E-02	=	2.34E-08	+	2.14E-08	+	2.40E-08	+	2.44E-08	+	2.40E-08	+
***Thoracic Surgery***	3.73E-09	+	2.34E-10	+	1.40E-08	+	1.00E+00	-	1.79E-09	+	2.83E-09	+	3.39E-09	+	2.34E-10	+
***Arcene Cancer***	1.55E-09	+	9.37E-10	+	2.07E-10	+	6.80E-10	+	3.78E-09	+	1.61E-09	+	6.76E-10	+	7.45E-07	+
***Colon***	7.13E-07	+	1.29E-09	+	7.13E-07	+	9.91E-01	-	7.13E-07	+	1.29E-09	+	1.29E-09	+	4.00E-07	+
***DLBCL***	2.22E-03	+	2.15E-03	+	NaN	=	9.73E-03	+	NaN	=	NaN	=	NaN	=	NaN	=
***Parkinson Ⅱ***	3.98E-09	+	3.98E-09	+	3.98E-09	+	7.52E-09	+	3.04E-08	+	3.98E-09	+	1.72E-08	+	1.21E-08	+
***Prostate Tumors***	1.96E-09	+	8.04E-02	=	NaN	=	NaN	=	4.26E-04	+	NaN	=	NaN	=	NaN	=
***Leukemia***	9.73E-03	+	NaN	=	NaN	=	NaN	=	NaN	=	NaN	=	3.76E-01	=	NaN	=
**+/ = /-**	15/0/1		12/3/1		12/4/0		11/3/2		13/2/1		12/3/1		13/3/0		11/3/2	

In summary, the performance evaluation on medical datasets demonstrates that BIMGO significantly outperforms the other optimization algorithms in terms of the fitness value and sensitivity. A 5% Wilcoxon rank-sum test verifies that BIMGO significantly outperforms other competing algorithms. Additionally, the BIMGO tends to select fewer features while maintaining optimal performance on most medical datasets. These results indicate the effectiveness of the BIMGO in addressing feature selection challenges in medical data.

#### 4) Parameter sensitivity analysis

In optimization problems, different parameter values may lead to different results. The BIMGO algorithm proposed in this paper to solve the feature selection problem for medical data does not have its own control parameters. Therefore, in this section, we discuss the effect of the population size and number of iterations on the BIMGO. We chose 5 datasets with different dimensions for our experiments, namely Diabetes, Glioma, Bone, Parkinson Ⅱ, and Arcene Cancer. The values of the parameters for the sensitivity analysis were set as follows: population size N = {20, 30, 50} and maximum number of iterations *MaxIter* = {50, 100, 200}. Each experiment was run independently 20 times. The parameter sensitivity was analyzed based on the average fitness values and convergence curves.

**Fig 8:**
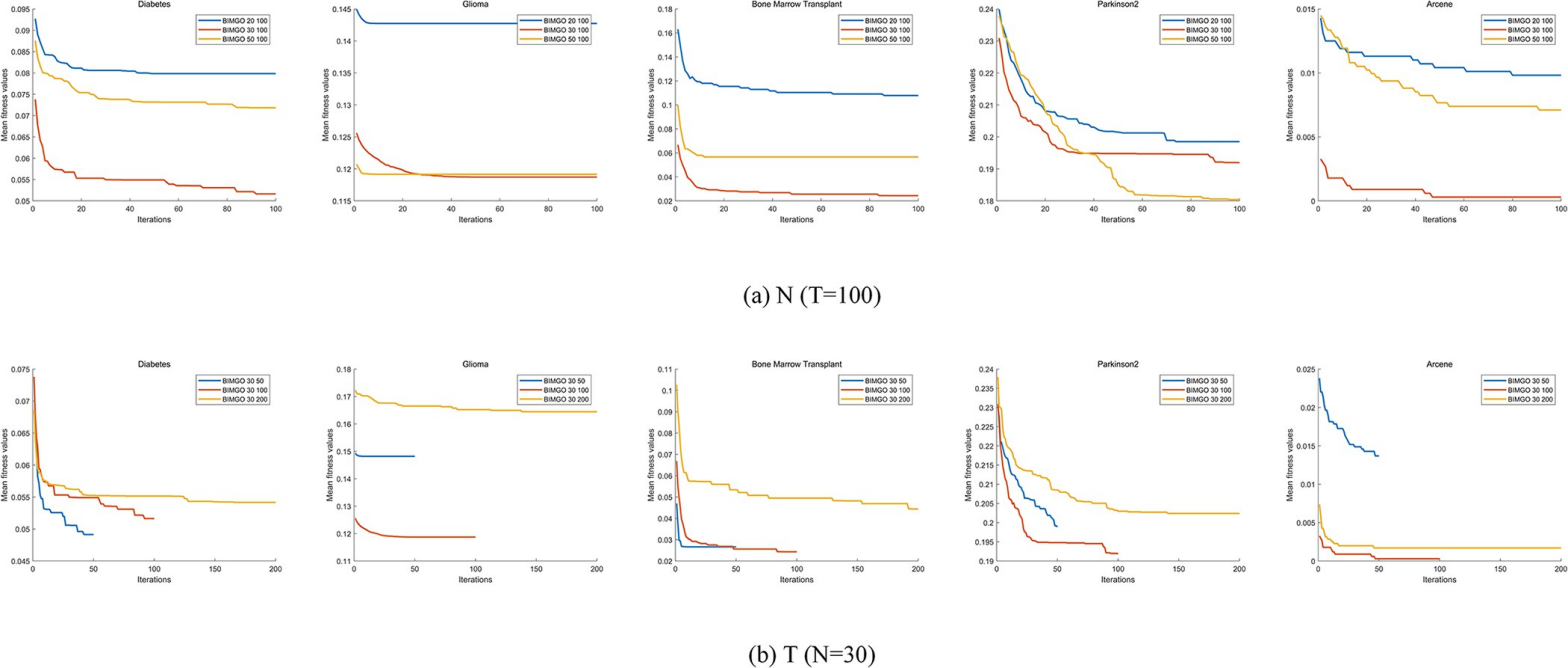
Parameter sensitivity of BIMGO.

Table 13 shows the average fitness values obtained by the BIMGO using different parameter values on 5 datasets. Fig 8 shows the convergence curves of the BIMGO for different population sizes and numbers of iterations.

**Table 13:** Mean fitness values for BIMGO using different parameters.

Parameter	*N*			*MaxIter*		
Value	20	30	50	50	100	200
***Diabetes***	0.0491	0.0516	0.0542	0.0798	0.0516	0.0718
***Glioma***	0.1482	0.1187	0.1644	0.1427	0.1187	0.1192
***Bone***	0.0266	0.0243	0.0444	0.1079	0.0243	0.0566
***Parkinson Ⅱ***	0.1989	0.1919	0.2024	0.1985	0.1919	0.1804
***Arcene Cancer***	0.0137	0.0003	0.0017	0.0098	0.0003	0.0071

The experimental results and the convergence curves indicate that the increase in population size does not improve the performance of the algorithm in solving feature selection problems. The appropriate selection of population size will affect the performance of the algorithm. As the number of iterations increases, the BIMGO continues to converge toward the optimal solution. The BIMGO has relatively low sensitivity to these parameters, and the differences between the results are relatively small among these parameters.

#### 5) Discussions

This paper presents an effective solution for feature selection tasks on medical datasets of different dimensions. The algorithm continuously searches in the solution space using four different mechanisms, and this powerful exploration capability enables the BIMGO algorithm to quickly search for a feasible solution during the feature selection process, especially for high-dimensional medical datasets. The introduction of the ICMIC in the initialization phase increases the diversity of the algorithm. The introduction of nonlinear control factors can balance exploration and exploitation and improve the search efficiency of the algorithm. In addition, the quality of feasible solutions is effectively improved by the spiral perturbation mechanism and the optimal individual neighborhood search strategy to locally search the current individual and the optimal individual in the iterative process, respectively, which is confirmed by the excellent performance on the evaluation criterion of the recall rate. The results of the evaluation comparison experiments indicate that the proposed BIMGO algorithm performs well in feature selection for medical data.

The advantages of the BIMGO include the following 6 points. First, the BIMGO generates solutions with better fitness values than those using competing algorithms in most cases, especially on high-dimensional datasets. Second, the BIMGO exhibits a stronger convergence ability than competitive algorithms do. The feature size is reduced by 66.39% on average on medical datasets with rich dimensionality, a result that is significantly better than that of the competitive algorithms. The BIMGO achieves the best performance in terms of recall, indicating that this algorithm has an excellent ability to select true positives, i.e., identifying disease features. This means that the BIMGO is suitable for feature selection tasks when processing medical data. Furthermore, it is clear from the statistical data analysis that the solutions generated by BIMGO are significantly better than those of other optimization algorithms in most cases. Finally, the design of the BIMGO algorithm is clear and easy to understand, and further enhancements performed on the subbasis will be easily implemented.

Although the results of the evaluation experiments indicate that the proposed BIMGO algorithm performs better in dealing with the FS problem for medical data, there are still some limitations. First, the BIMGO algorithm exhibits average performance on low-dimensional datasets, and it cannot select fewer features on these datasets. Second, the computational cost of the BIMGO is high and is not applicable to applications that are sensitive to computational cost. The SVM was chosen as the learning scheme in this study. Although the SVM exhibited better accuracy, it has a high time cost and is sensitive to noisy data. Finally, in this study, only feature selection for medical datasets was performed, and the application of the BIMGO to different domains and problem types needs further discussion and research.

## V. Conclusion and future works

To address the feature selection problem, we proposed an improved IMGO based on the MGO algorithm and its binary version, namely, the BIMGO. To improve the algorithm’s optimization ability, the proposed algorithm uses four new strategies: applying the ICMIC to initialize the population, introducing nonlinear control factors to control the search process, using a spiral perturbation mechanism to perturb the individuals in each iteration, and designing the neighborhood search strategy for the optimal individual. The use of these strategies can improve the diversity of the population, enhance the development and convergence ability, and improve the search efficiency and the quality of the solution of the algorithm. Two sets of experiments were conducted to verify the ability of the IMGO to address continuous problems and the BIMGO to address feature selection problems. In the first set of experiments, 23 benchmark functions were used to compare the IMGO algorithm with competitive algorithms, and the experimental results showed that the IMGO algorithm effectively outperformed the competitive algorithms as IMGO achieved the best results on 18 benchmark functions. In the second set of experiments, we evaluated the performance of the BIMGO on 16 medical datasets with dimensions ranging from 10 to 10509 and compared it with that of 8 well-known metaheuristic algorithms in terms of the fitness value, number of selected features, and sensitivity. The experimental results show that BIMGO achieved the best fitness values on 10 datasets, and its overall performance is the best among all algorithms. In terms of sensitivity, BIMGO achieved the best results on 10 datasets and the overall performance. The performance of BIMGO in sensitivity indicates its applicability in processing medical data. BIMGO is able to select as few features as possible while ensuring accuracy, and its average feature approximation rate is 66.39%. The experimental results showed that the BIMGO significantly outperformed the compared algorithms in most cases. Finally, the experimental results were statistically analyzed using the Wilcoxon rank-sum test, which indicated that the BIMGO significantly outperformed the competing algorithms in handling the feature selection problem for processing medical data.

However, the BIMGO still has several limitations: its performance on low-dimensional datasets is mediocre, and its computational cost is high. In the future, we will further improve the algorithm and conduct in-depth research on its performance in combination with different classifiers. Moreover, in addition to comparisons with classical algorithms, we will also pay attention to other novel metaheuristic algorithms that can be used as comparison algorithms to further evaluate the performance of the BIMGO and to improve its performance in the future. In addition, we would like to apply the algorithm to other fields, such as finance and engineering.

## Supporting information

S1 Dataset (ZIP)
